# Noncovalent Targeting of Nanocarriers to Immune Cells with Polyphosphoester‐Based Surfactants in Human Blood Plasma

**DOI:** 10.1002/advs.201901199

**Published:** 2019-10-04

**Authors:** Johanna Simon, Kristin N. Bauer, Jens Langhanki, Till Opatz, Volker Mailänder, Katharina Landfester, Frederik R. Wurm

**Affiliations:** ^1^ Max‐Planck‐Institut für Polymerforschung Ackermannweg 10 55128 Mainz Germany; ^2^ Dermatology Clinic University Medical Center of the Johannes Gutenberg‐University Mainz Langenbeckstr. 1 55131 Mainz Germany; ^3^ Institute of Organic Chemistry Johannes Gutenberg‐University Mainz Duesbergweg 10‐14 55128 Mainz Germany

**Keywords:** dendritic cells, protein corona, ring‐opening polymerization, stealth effect, targeted drug delivery

## Abstract

Dendritic cells (DCs) are part of the immune system and can internalize pathogens by carbohydrate receptors. The uptake induces maturation and migration of the DCs resulting in an adaptive immune response by presenting antigens to T‐cells. Thus, targeted delivery to DCs is a powerful tool for immunotherapy. However, in blood, specific targeting is challenging as blood proteins adsorb to the nanocarriers and mask the targeting molecules. Additionally, covalent coupling of targeting groups to nanocarriers requires new chemistry for each nanocarrier, while a general strategy is missing. A general protocol by noncovalent adsorption of mannosylated polyphosphoesters (PPEs) on the nanocarriers' surface resulting in specific uptake into DCs combined with low protein adsorption of PPEs is presented. PPEs with hydrophobic anchors and multiple mannose units are reported and adsorbed to different model nanocarriers. Their protein corona remain similar to pure stealth nanocarriers and prove only low uptake into nontargeted cells (monocytes). Due to the “stealth” properties of PPEs, a high specific uptake into DCs is achieved after incubation in human blood plasma, proving an efficient combination of “stealth” and targeting after simple adsorption of the PPEs. This strategy can transform any nanocarrier into DC‐targeting by noncovalent adsorption of PPEs and will aid in developing novel immunotherapies.

## Graphical abstract

Mannose‐functionalized polyphosphoesters (PPEs) combine stealth properties with selective cellular uptake. Polymer nanocarriers are noncovalently coated with PPEs and their selective uptake in dendritic cells (DCs) is studied. The carbohydrate functionalization in the pendant chains of the PPEs leads to a receptor‐mediated uptake into DCs, while the stealth effect of PPEs prevents an unselective uptake in other cell types.

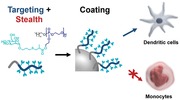

## 1 Introduction

Targeting of nanocarriers in the human bloodstream to specific cell types is a major challenge for modern drug delivery.^1^ Dendritic cells (DCs) are the most effective antigen‐presenting cells and occupy a pivotal role in initiating T‐cell‐mediated immunity.^2^, ^3^ As guardians of the immune system, they are specialized for the recognition and uptake of pathogens. Receptor‐mediated uptake of pathogens into immature DCs leads to their maturation and migration to lymphoid organs in which they activate T‐cells. DCs process microbial antigens and present them to resting T‐cells, thereby initiating adaptive immune responses. Recent strategies in the development of therapeutic vaccines have focused on the ability to deliver antigens to DCs, which can then trigger the desired T‐cell function.^4^ The delivery of nanocarriers to DCs while preventing unspecific cellular uptake by other cells remains a challenge.^5^

Here, we present the selective uptake of polymeric nanocarriers to monocyte‐derived DCs (moDCs) by noncovalently attached polyphosphoesters (PPE) carrying mannose targeting units. To date, the targeting ligands (e.g., an antibody or carbohydrate) had been covalently attached to the nanocarriers.[qv: 6b,7] Covalent attachment of the targeting moieties often requires considerable synthetic efforts and is additionally plagued by low degrees of functionalization and the chemistry needs to be designed for each nanocarrier system.^8^, ^9^ In contrast, a noncovalent attachment of targeting groups by adsorption allows transforming basically any material into a targeting nanocarrier.^10^ Mannose was chosen as a targeting ligand to enable cellular recognition by monocyte‐derived dendritic cells.^11^, ^12^

However, designing nanocarriers with specific cell targeting for in vivo application bears additional challenges.^13^ Once the nanocarriers are introduced to the bloodstream, proteins rapidly cover their surface and critically alter their physicochemical properties.[qv: 14b] Several reports recognized that targeting ligands were completely buried by blood proteins and specific cellular interactions were prevented.[qv: 6a,15] In order to minimize the protein adsorption, we used polyphosphoesters to coat the nanocarriers' surface. PPEs are currently discussed as promising materials in biomedical applications,^16^ as they have a versatile chemical structure,^17^ are biodegradable, and proved to suppress unspecific cellular uptake (of PPEylated nanocarriers).^18^ The PPE platform allowed us to prepare amphiphilic and mannosylated PPEs that were adsorbed on the surface of different nanocarriers. We were able to prove that the remaining protein corona on the PPEylated nanocarriers did not reduce the targeting ability of the mannose‐functionalized nanocarriers. Additionally, we carried out a detailed proteomic investigation to identify the distinct protein corona pattern underlining the combination of stealth properties together with specific targeting. This strategy proves the efficient combination of stealth and targeting properties that can be installed into different nanocarriers via coating with polyfunctional PPEs.

## 2 Results and Discussion

**Figure 1:**
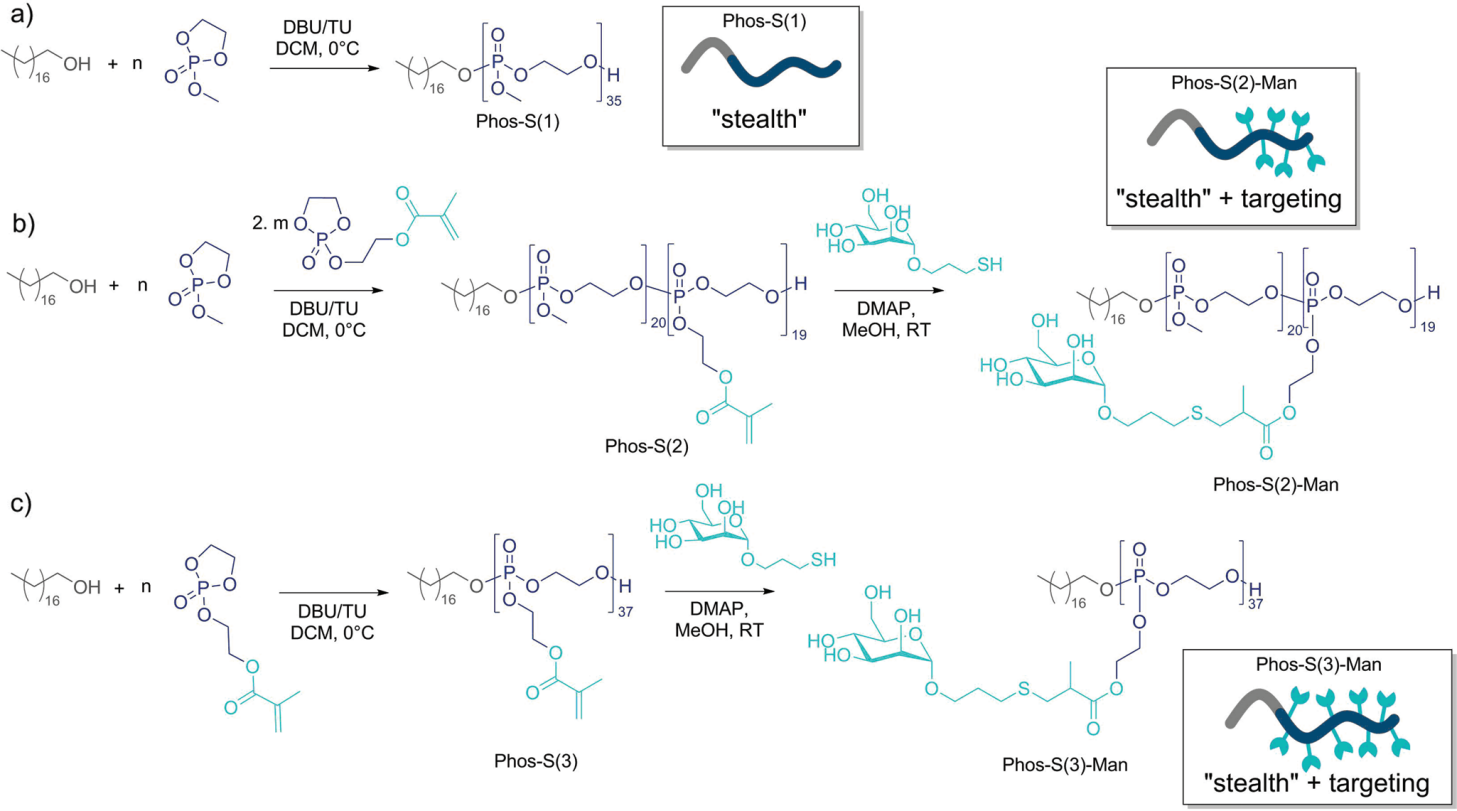
Synthesis of polyphosphoester amphiphiles by ROP of cyclic phosphoester monomers and subsequent mannosylation by thiol–Michael addition. a) Preparation of Phos‐S(1) without mannose moieties (for stealth behavior). b) Synthesis of block copolymer Phos‐S(2) and subsequent mannosylation leading to Phos‐S(2)‐Man. c) Synthesis of Phos‐S(3) by ROP of OPEMA and subsequent mannosylation by thiol–Michael addition leading to Phos‐S(3)‐Man.

The mannosylated amphiphilic PPEs were designed to consist of a stearyl alcohol as the hydrophobic anchor and a hydrophilic PPE segment that was functionalized with several mannose units. The amphiphiles were prepared by the organocatalyzed ring‐opening polymerization (ROP)[qv: 18b] of two different cyclic phosphoester monomers, that is, 2‐methoxy‐1,3,2‐dioxaphospholane 2‐oxide (MEP)^19^ and the side‐chain functional 2‐((2‐oxido‐1,3,2‐dioxaphospholan‐2‐yl)oxy)ethyl methacrylate (OPEMA).^20^ PMEP was chosen as a hydrophilic PPE, while P(EPEMA) was chosen to attach thiol‐modified mannose to the pendant methacrylates by a quantitative nucleophilic addition. Three different PPE amphiphiles with different amounts of reactive groups were prepared: Phos‐S(1) was based on PMEP only, Phos‐S(2) was a block copolymer (with 19 methacrylate units), and Phos‐S(3) was a homopolymer of OPEMA (with 37 methacrylate units, **Figure** **1**). The P(EPEMA) units are water‐insoluble intermediates, but after mannosylation the PPEs were water‐soluble amphiphiles with surface tensions of 47.34 ± 0.07 mN for Phos‐S(2)‐Man and 51.21 ± 0.03 mN for Phos‐S(3)‐Man (*c*_amphiphile_ = 1 g L^−1^).

In all cases, stearyl alcohol was used as the initiator and the polymerization was catalyzed by 1,8‐diazabicyclo[5.4.0]undec‐7‐ene/thiourea (DBU/TU) as previously reported for other cyclic phospholanes.^21^ The respective NMR spectra and size‐exclusion chromatography (SEC) analyses are found in Figures S1–S22 (Supporting Information). The monomer to initiator feed ratio allowed the adjustment of the number of functional groups in the PPE amphiphiles. All polymers exhibited moderate molar mass dispersities 1.2 < *Ð* < 1.5 and molar masses between 5200 and 9000 g mol^−1^ (Table S1, Supporting Information). Phos‐S(2) and Phos‐S(3) were reacted with mercaptopropyl‐α‐d‐mannopyranoside (2), resulting in the mannosylated amphiphiles Phos‐S(2)‐Man (*M*_n_(NMR) = 12 300 gmol^−1^) and Phos‐S(3)‐Man (*M*_n_(NMR) = 18 400 gmol^−1^), carrying 19 or 37 mannose moieties, respectively (Table S2, Supporting Information).

**Figure 2:**
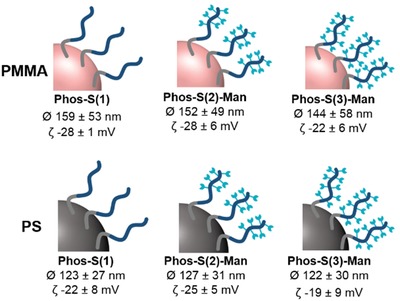
Characteristics of polystyrene and poly(methyl methacrylate) nanocarriers coated with PPE amphiphiles.

To study the efficiency of these targeting amphiphiles for the receptor‐mediated uptake into monocyte‐dervived dendritic cells (moDCs), two different model nanocarriers (NCs) were prepared by free‐radical miniemulsion polymerization of styrene or methyl methacrylate. The model nanocarriers had diameters of 101 ± 12 nm for polystyrene (PS) and 114 ± 15 nm for poly(methyl methacrylate) (PMMA, Table S3, Figures S23 and S24, Supporting Information). The PPE‐amphiphiles were subsequently adsorbed to the surface of the PS or PMMA nanocarriers. The dispersions were characterized by dynamic light scattering (DLS) after the coating process proving that there was no aggregation in water (**Figure** **2**). To underline the colloidal stability in human blood plasma, DLS of all PPE‐coated NCs was conducted after incubation with blood plasma. There was no significant size increase before and after incubation with plasma (Figure S25, Supporting Information). Additionally, there were no cytotoxic effects for PPE‐coated NCs (Figure S26, Supporting Information).

**Figure 3:**
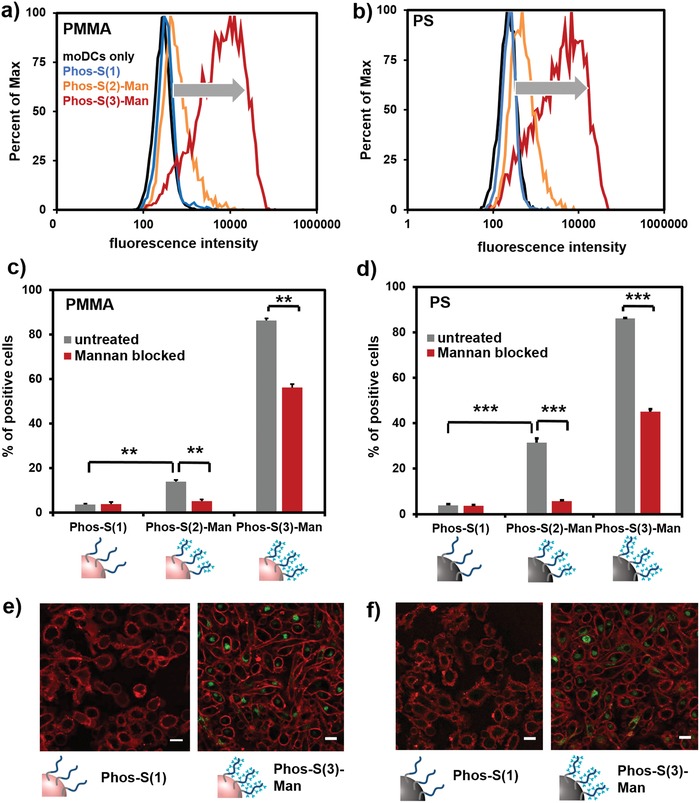
Interactions of PPEylated nanocarriers with dendritic cells. a,b) Flow cytometry analysis: untreated cells (black), Phos‐S(1) (blue), Phos‐S(2)‐Man (orange), and Phos‐S(3)‐Man (red)‐coated NCs (PS = polystyrene, PMMA = poly(methyl methacrylate)). c,d) Dendritic cells were incubated with NCs (150 µg mL^−1^) for 2 h, 37 °C. Cellular uptake was quantified by flow cytometry and values are expressed % positive cells ± SD from triplicates (gray bars). For blocking experiments, cells were pretreated with mannan (3 mg mL^−1^) for 30 min, 4 °C (red bars). e,f) Representative CLSM of dendritic cells incubated with PPE‐coated NCs (150 µg mL^−1^) for 2 h, 37 °C. The cell membrane was stained with CellMask Orange and is pseudo‐colored in red whereas the NCs were pseudo‐colored in green. Scale bar: 20 µm.

In order to evaluate whether the coating of NCs with mannose‐functionalized PPE amphiphiles allows receptor‐mediated cellular uptake, flow cytometry analysis toward moDCs was conducted (**Figure** **3**a,b). Flow cytometry analysis and confocal laser microscopy images (Figure 3e,f and Figure S27, Supporting Information) confirmed that both NCs coated with Phos‐S(1) (no mannose) exhibit a low binding affinity toward moDCs indicating the stealth properties of a PPE‐modified nanocarrier. In contrast, for both PS and PMMA nanocarriers, coated with mannose‐functionalized PPEs (Phos‐S(2)‐Man or Phos‐S(3)‐Man), we proved a significant increase in their cellular uptake into DCs (Figure 3c,d). This increased cellular uptake of the nanocarriers is already a good indication that the noncovalent adsorption of mannosylated PPEs on the NCs led to an increased receptor‐mediated uptake into the DCs. Additionally, we observed that NCs coated with Phos‐S(3)‐Man displayed a significantly higher cellular internalization compared to NCs coated with Phos‐S(2)‐Man. As the mannose–lectin interaction is a cooperative effect, this difference in cellular uptake might be directly linked to the higher amount of mannose units in Phos‐S(3)‐Man compared to Phos‐S(2)‐Man. To prove this hypothesis and to investigate the accessibility of the mannose unit on the nanocarriers surface, a lectin binding assay was performed. Therefore, NCs were incubated with a fluorescently labeled lectin, which specially recognizes mannose units. If the lectin binds to the mannose unit on the nanocarriers' surface, this newly formed complex can be detected via flow cytometry. Here, a significantly higher amount of mannose on the surface of Phos‐S(3)‐Man‐coated NCs compared to Phos‐S(2)‐Man was detected (Figure S28, Supporting Information).

In order to verify a CD206/CD209 receptor‐mediated uptake of the mannose‐modified NCs, blocking experiments were performed (Figure 3c,d). Cells were pretreated with mannan, which strongly binds to the mannose receptor and blocks it for further docking of the NCs. Blocking with mannan proved a strong reduction of the internalization of all mannose‐modified NCs, as the mannose receptor could not further recognize the mannose units on the PPEylated NCs (*p* < 0.001***). These experiments clearly prove that the prepared PPE increased a receptor‐mediated uptake of nanocarriers into DCs. As a complementary approach, the mannose receptor was blocked with soluble antibodies (Figure S29, Supporting Information). If cells were treated with antibodies prior to uptake analysis, cellular interactions of NCs coated with mannose PPE amphiphiles were also reduced, proving the receptor‐mediated uptake of mannose‐PPEylated NCs.

**Figure 4:**
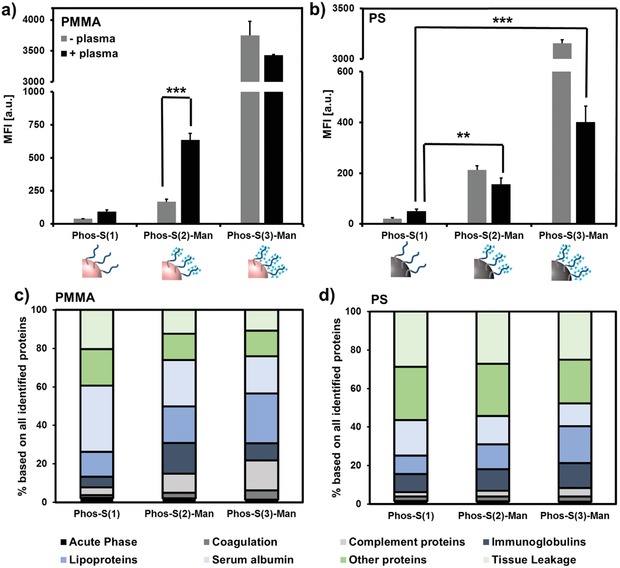
a,b) Cell interactions of NCs after blood incubation: PPE‐coated PS or PMMA NCs were exposed to human blood plasma and the cellular uptake (150 µg mL^−1^, 2 h) toward dendritic cells was quantified via flow cytometry. Values are expressed as mean ± SD from triplicates. c,d) Protein corona analysis from mass spectrometry based proteomics. All identified proteins were classified into eight different protein classes depending on their biological function. A full list of all detected proteins is summarized in a separate Excel Sheet (Table S4, Supporting Information).

The previous cell experiments confirmed the targeting under artificial conditions in cell culture medium, but in the absence blood plasma proteins. The loss of specific targeting after blood incubation had been reported as plasma proteins tend to bury the targeting groups.[qv: 6a,13] Also in our case, after incubation into human blood plasma, the cellular uptake of the nanocarriers was influenced by the protein corona: Flow cytometry experiments indicated that PS nanocarriers, which were coated with both mannosylated amphiphiles, exhibited a lower cell internalization after the protein corona formation compared to the protein‐free scenario (**Figure** **4**b). This effect was more pronounced for Phos‐S(3)‐Man compared to Phos‐S(2)‐Man, probably due to the overall stronger cell uptake in the former case (*p* < 0.001***). However, a significantly higher amount of the mannosylated NCs was still selectively taken up into the DCs as compared to Phos‐S(1)‐coated NCs. The low protein adsorption onto PPEylated surfaces still guaranteed access to the mannose units and resulted in a cellular uptake into the DCs. As PPEs were recently identified by our group as potential stealth polymers that reduce unspecific cell uptake by selected adsorption of lipoproteins,^22^ we conducted a detailed proteomic analysis of the mannose‐modified PPEylated NCs. All identified proteins were classified into eight different classes depending on their biological function (Figure 4c,d). In general, the amount of adsorbed protein and the overall corona composition were very similar for the mannosylated and non‐mannosylated NCs (Figure S30, Supporting Information). The corona of Phos‐S(3)‐coated PS NCs was enriched with lipoproteins (19%) compared to NCs coated with Phos‐S(1) (9%) or Phos‐S(2) (13%) (Figure 3d, and Figures S31–S33, Supporting Information). In previous studies, we found that lipoproteins, in particular clusterin, interacted with PEGylated and PPEylated NCs and reduced the cellular interactions with macrophages.[qv: 22a] Their presence in the corona explains the reduced uptake and indicates that corona proteins can shield the targeting ligand, but do not suppress the interaction with the mannose receptor. Interestingly, the internalization of the PMMA NCs seemed to be relatively unaltered in the presence of blood proteins (Figure 4a). We even detected an enhanced cellular uptake (*p* < 0.001***) for PMMA NCs coated with Phos‐S(2)‐Man after corona formation. This effect can be explained by the distinct corona composition. We detected a higher amount of immunoglobulins (15%) on the surface of Phos‐S(2)‐Man‐coated NCs compared to Phos‐S(1)‐Man (6%) or Phos‐S(3)‐Man ones (9%). Immunoglobulins are referred to the class of opsonins, which are known to enhance interaction with immune cells.^23^ Therefore, the interaction of Phos‐S(2)‐Man‐coated PMMA NCs and moDCs could be favored.

**Figure 5:**
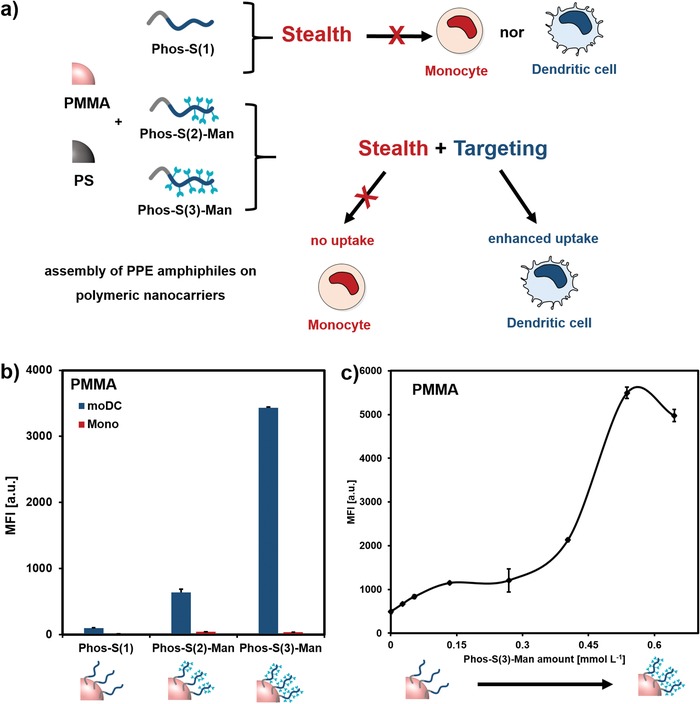
a) PPE amphiphiles adsorbed on polymer nanocarriers possess stealth and targeting properties. b) PMMA nanocarriers were exposed to human blood plasma and cellular uptake (150 µg mL^−1^, 2 h) toward dendritic cells (blue) or monocytes (red) was quantified via flow cytometry. Values are expressed as mean ± SD from triplicates. c) PMMA nanocarriers were incubated with different amounts of mannoslyated PPE‐amphiphile. Cellular uptake toward moDCs (150 µg mL^−1^, 2 h) of Phos‐S(3)‐coated nanocarriers was analyzed via flow cytometry after plasma coating. Values are expressed as mean ± SD from duplicates.

The specificity of the mannosylated‐NCs for DCs was further underlined by incubation with monocytes. Monocytes are one type of precursor cells of DCs and are the first cells, which recognize invading pathogens. However, they only express low levels of the mannose receptor (CD206/CD209, Figure S34, Supporting Information). As expected, the non‐mannosylated PPEylated NCs were also not taken up by the monocytes. More importantly, the mannosylated NCs only exhibited a very low internalization into monocytes, which further underlines their specific interaction with the mannose receptor of dendritic cells even in the presence of the protein corona (PMMA: **Figure** **5**b and PS: Figure S35, Supporting Information).

Finally, we investigated the influence of the mannose density on the targeting efficiency. Therefore, PMMA nanocarriers were coated with different amounts of Phos‐S(3). Here, we found that there was no linear correlation between the mannose amount and the cell uptake behavior (Figure 5c). Upon a certain threshold, the cellular uptake significantly increased. As described, the mannose receptor (CD206/CD209) specifically recognized multivalent mannose units meaning that the NCs need to be coated with a high mannose density.^24^ In the case of Phos‐S(2), the cell interaction was lower compared to Phos‐S(3) (Figures 4 and 5). This goes along with a slightly lower mannose density. As initially investigated via the lectin binding assay, there was also a higher accessibility of the mannose for Phos‐S(3)‐coated NCs compared to Phos‐S(2)‐coated NCs (Figure S28, Supporting Information).

## 3 Conclusion

The control of the protein adsorption from human blood plasma is crucial to establish specific cell targeting of nanocarriers. Herein, we adsorbed amphiphilic and mannosylated polyphosphoesters on the surface of model polymeric nanocarriers. The combination of the stealth properties of the polyphosphoester with the additional possibility to attach targeting units to the pendant phosphoester proved an effective strategy to achieve the receptor‐mediated uptake into dendritic cells of the immune system even after contact with human blood. Additionally, an overall low uptake in nontargeted cells (monocytes) was found. Therefore, the here presented approach allows the targeting of any nanocarrier into dendritic cells by simply adsorption of the mannosylated PPE‐amphiphiles on their surface. This is a step toward understanding and controlling the behavior of functional drug delivery vehicles in blood.

## Conflict of Interest

The authors declare no conflict of interest.

## Supporting information

Supplementary
