# Supplementary material for: Noncovalent Targeting of Nanocarriers to Immune Cells with Polyphosphoester‐Based Surfactants in Human Blood Plasma
Source: Adv Sci (Weinh). 2019 Oct 4;6(22):1901199. doi: 10.1002/advs.201901199 (PMC6864500; doi:10.1002/advs.201901199)
Supplement: Supplementary file 1 — Supplementary [file ADVS-6-1901199-s001.pdf]

Copyright WILEY-VCH Verlag GmbH & Co. KGaA, 69469 Weinheim, Germany, 2019.

ADVANCED SCIENCE
Open Access

Supporting Information

for *Adv. Sci.,* DOI: 10.1002/advs.201901199

Noncovalent Targeting of Nanocarriers to Immune Cells with Polyphosphoester-Based Surfactants in Human Blood Plasma

*Johanna Simon, Kristin N. Bauer, Jens Langhanki, Till Opatz, Volker Mailänder,**
Katharina Landfester, and Frederik R. Wurm**

Supporting Information

Noncovalent targeting of nanocarriers to immune cells with polyphosphoester-based surfactants in human blood plasma

Johanna Simon[a,b,1], Kristin N. Bauer[a,1], Jens Langhanki[c], Till Opatz[c], Volker Mailänder[b,a,*], Katharina Landfester[a,*], Frederik R. Wurm[a,*]

[a] Max-Planck-Institut für Polymerforschung, Ackermannweg 10, 55128 Mainz, Germany. wurm@mpip-mainz.mpg.de

[b] Dermatology Clinic, University Medical Center of the Johannes Gutenberg-University Mainz, Langenbeckstr. 1, 55131 Mainz, Germany.

[c] Institute of Organic Chemistry, Johannes Gutenberg-University Mainz, Duesbergweg 10-14, 55128 Mainz, Germany.

[1] Shared first author

* Senior and corresponding authors

Content

1. Material and methods 3

2. Synthetic procedures 6

   2.1 Carbohydrate synthesis 6

   2.2 Surfactant synthesis 8

   2.3 Nanoparticle Preparation 12

   2.4 NMR Spectra 13

   2.5 SEC 22

3. Colloid stability and toxicity 24

4. Confocal laser scanning microscopy 26

5. Detection of mannose on the nanocarrier surface 26

6. Antibody blocking 27

7. Influence of protein corona formation 28

8. Expression level of the mannose receptor 30

7.References 30

1. Material and methods

General Information. All chemicals and solvents were purchased from Sigma-Aldrich, Acros Organics or Fluka and used as received unless otherwise stated. Deuterated solvents were purchased from Sigma-Aldrich and used as received. The eluents for column chromatography (cyclohexane and ethyl acetate) were distilled prior to use. Deutero chloroform was stored over alumina (Brockmann activity I).

SEC. For the size exclusion chromatography (SEC) measurements two different methods were used. ***Method a***: SEC measurements of standard polymers were performed in DMF (containing 1 gL^{-1} of lithium bromide as an additive) at 60 °C and a flow rate of 1 mL min^{-1} with a PSS SECurity as an integrated instrument, including a set of 3 PSS GRAM columns (porosity of 100 Å and 1000 Å) and a refractive index (RI) Detector. Calibration was carried out using polyethylene glycol standards provided by Polymer Standards Service. ***Method b***: SEC measurements were performed in DMF (containing 1 gL^{-1} of lithium bromide as an additive) at 50 °C with an Agilent 1100 Series as an integrated instrument, including a HEMA column (300/100/40) from MZ Analysentechnik, a UV (275 nm), and a refractive index (RI) detector. Calibration was carried out using poly(ethylene glycol) standards provided by Polymer Standards Service.

NMR. NMR spectra of the mannose derivatives **(2)** and **(3)** were recorded on an *Avance II 400* (400 MHz ^{1}H NMR, 101 MHz ^{13}C NMR, COSY, HSQC, HMBC; *Bruker*), using a 5 mm probe head at a temperature of 23°C. The ^{13}C-NMR spectra are ^{1}H broadband decoupled. The HSQC sepctra are phase-sensitive (opposite signs for CH/CH_3 and CH_2).The ^{1}H and ^{13}C chemical shifts (δ) were referenced to the residual solvent signal as internal standard (DMSO-d_6: δ = 2.50 ppm and 39.52 ppm, CD_3OD: δ = 3.31 ppm and 49.00 ppm for ^{1}H and ^{13}C NMR, respectively).[1] Coupling constants (*J*) are reported in Hz (splitting abbreviations: s, singlet; d, doublet; t, triplet; q, quartet; m, multiplet; br, broad; and combinations thereof). ^{1}H, ^{13}C and ^{31} P NMR spectra of mannose derivative **(3)** and all polymers were recorded using a Bruker Avance III 250, a Bruker Avance 300 or a Bruker Avance III 500 and processed with MestReNova. All ^{31}P NMR spectra were recorded decoupled from protons. All spectra were referenced internally to residual proton signals of the deuterated solvent. 2D spectra were processed with the topspin 3.5 software.

For the diffusion measurements (DOSY)[2] a 5 mm triple resonance BBFO $^{1}H/X$ probe equipped with a z-gradient on the 500 MHz Bruker AVANCE III system was used. For the diffusion measurements a 2D sequence (DOSY, dstebpgp3s) with a stimulated echo was

used.[3] The temperature was kept at 298.3 K and regulated by a standard ^1H methanol NMR sample using the topspin 3.5s software (Bruker). The control of the temperature was realized with a VTU (variable temperature unit) and an accuracy of +/- 0,1K. The diffusion time was 30-70 ms and the gradient length to 1.4 ms. The relaxation delay between scans was 1.5-2 s. The gradient strength was calibrated by analysis of a sample of $2H_2O/1H_2O$ at a defined temperature and comparison with the theoretical diffusion coefficient of $2H_2O/1H_2O$.

Cell culture. Monocytes and monocyte derived dendritic cells were isolated from human buffy coats according to the vote of the local ethics committee and the Declaration of Helsinki as previously reported[4]. Briefly, peripheral blood mononuclear cells (PBMCs) were isolated by Ficoll separation and plated into 6-well plates in DC-medium (RPMI + 2% human serum, 100 IU/mL penicillin, 100 µg/mL streptomycin). Cells were incubated for 1 h at 37 °C and non-adherent cells were further discarded by washing the plates with PBS (3-4 times). Adherent cells were either directly frozen and further used for monocyte experiments or differentiated into monocyte derived dendritic cells (moDCs). For generate moDCs, cells were incubated with 800 IU/mL granulocyte-macrophage colony-stimulating factor (GMCSF) and 500 IU/mL interleukin IL4 (both PromoCell). Afterwards 48 h the medium was refreshed with 1600 IU/mL GMCSF and 500 IU/mL IL4 and incubation for 72 h was followed. MoDCs were detached from the wells with 0.5 mM PBS-EDTA solution.

Surface marker. 100 000 cells were resuspended in 100 µL PBS and the following antibodies were used to label the surface marker for the mannose receptor (APC anti-human CD206 MMR Antibody (Biolegend) and APC anti-human CD209 MMR Antibody (Biolegend)). Antibodies (5 µL) were added to cells for 30 min, 4 °C. Surface marker expression was analyzed by flow cytometry via Attune NxT Flow Cytometer.

Cell uptake by flow cytometry. 100 000 cells were seeded in DC medium with human serum into 48-well and incubated overnight at 37 °C. Nanocarriers (150 µg/mL) coated with or without plasma proteins (see below protein corona preparation) were added to DC medium without human serum and incubated with cells for 2 h, 37 °C. Cells were detached with 0.5 mM PBS-EDTA, centrifuged (480 g, 5 min) and resuspended in PBS. Samples were analyzed by flow cytometry via Attune NxT Flow Cytometer.

For blocking studies: Mannan (3 mg/mL) was dissolved in DC medium without human serum and added to cells for 30 min, 4 °C. For further blocking studies, mannan was kept in the cell culture medium during the incubation of cells and nanoparticles. Antibodies (anti-CD206/anti- CD209 concentration:25 µg/mL) were dissolved in DC medium without human serum and added to cells for 30 min, 4 °C. For further blocking studies, the antibodies were kept in the cell culture medium during the incubation of cells and nanoparticles.

Statistical analysis: An unpaired student´s t-test assuming equal variances was performed. The p values were defined as followed: $*p < 0.05$, $**p < 0.01$, $***p < 0.001$. Not significant differences are labelled as n.s.

Confocal laser scanning microscopy. To proof the intracellular localization of the nanoparticles, cell uptake experiments were conducted in the same manner as described for flow cytometer experiments. Images were taken with a LSM SP5 STED Leica Laser Scanning Confocal Microscope (Leica). Nanocarriers (BODIPY) were excited with an argon laser (514 nm) and the cell membrane was stained with CellMaskOrange (2.5 µg/mL) which was exited with a DPSS 561 nm laser. Nanocarriers are pseudo-colored in green and the cell membrane is pseudo colored in red.

Lectin assay. To detect mannose on the surface of the nanocarriers (1 µg) were incubated with Wheat Germ Agglutinin (WGA, Thermo) conjugated to Texas Red (1 µg) for 30 min in the dark. Nanocarriers with bound WGA were detected by flow cytometry.

Human plasma. Human plasma was obtained from the Department of Transfusion Medicine Mainz from healthy donors. A plasma pool from ten volunteers was used and storaged at - 80 °C.

Protein corona preparation. Nanocarriers (0.05 m^2) were incubated with 1 mL of human plasma for 1 h. Hard corona coated nanocarriers were isolated via centrifugation (20 000 g, 1 h, 4 °C) and washing with PBS (3 times, 1 mL). For cell uptake studies, nanocarriers were washed one time and added to cell culture medium.

To analyze the protein pattern surrounding the nanoparticles, the pellet was resuspended in 2% SDS (62.5 mM Tris*HCl) and heated up to 95 °C for 5 min. The dispersion was centrifuged (20 000 g, 1 h, 4 °C) again and the supernatant was analyzed by Pierce Assay, SDS-PAGE and LC-MS.

SDS-PAGE. Protein solutions (1 µg in 26 µL) were mixed with 4µL of NuPage Reducing Agent and 10µL of NuPage LDS Sample Buffer NuPage The sample was heated up to 70 °C for 10 min and loaded on a 10% Bis-Tris-Protein Gels using NuPAGE MES SDS Running Buffer (Thermo Fisher Scientific). The gel was run for 1 h at 100 V and the band were visualized by Silver Staining using the commercial SilverQuest Silver Staining Kit.

Protein quantification. The protein concentration was determine by Pierce 600 nm protein Assay according to the manufactures instruction.

In solution digestion. To remove SDS, protein solutions were applied to Pierce detergent removal columns (Thermo Fisher). Digestion was performed to previously estabilished

protocols.[5],[6] A protein:trypsin ratio of 50:1 was chosen and the reaction was carried out over 14 h at 37°C.

Liquid chromatography coupled to mass spectrometry (LC-MS analysis). Peptide samples were diluted with with 0.1% formic acid. Hi3 Ecoli was added at a concentration of 50 fmol/µL for absolute protein quantification[7]. A nanoACQUITY system containing a C18 analytical reversed phase column (1.7 µm, 75 µm × 150 mm) and a C18 nanoACQUITY trap column (5 µm, 180 µm × 20 mm) was used. Mobile phase A consists of 0.1% (v/v) formic acid in water and mobile phase B of 0.1% (v/v) formic acid with acetonitrile. For separation a gradient of 2% to 37% of mobile phase B over 70 min was used. The UPLC system was coupled to a Synapt G2- Si mass spectrometer performing electrospray ionization (ESI) in positive ion mode using a NanoLockSpray source. Glu-Fibrinopeptide was infused as reference component. The mass spectrometer was operated in resolution mode and d data-independent acquisition (MS^E) experiments were carried out. Data was processed with MassLynx 4.1

Protein identification. To identify proteins, a reviewed human data base (Uniprot) was downloaded and analysis was carried out with Progenesis QI (2.0). Parameters for protein identification were set as described before[8]. The human data base was modified with the sequence information of Hi3 Ecoli standard for absolute protein quantification. The amount of each protein in fmol was obtained via the TOP3/Hi3 approach[9].

2. Synthetic procedures

2.1 Carbohydrate synthesis

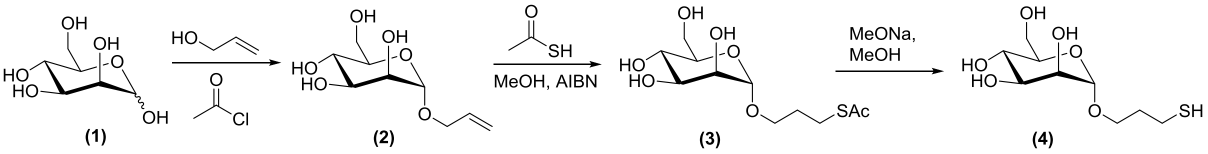

Allyl α-D-mannopyranose (2). A solution of α-D-mannopyranose (**1**, 5.00 g, 27.8 mmol, 1 eq.) in allylalcohol (48.4 g, 56.9 mL, 833 mmol, 30 eq.) was treated with acetyl chloride (5.41 g, 4.92 mL, 69.4 mmol, 1.5 eq.) under stirring at room temperature. The reaction mixture was heated to 100 °C and stirred at that temperature 16 hours. Subsequently the reaction mixture was cooled to room temperature and treated with solid $NaHCO_3$ until pH = 7. The mixture was filtered over a bed of Celite, which was washed thoroughly with toluene. The solvents were removed under reduced pressure and the residue was purified by flash column chromatography (ethyl acetate/ methanol, 10:1) to give the title compound (4.75 g, 21.6 mmol, 78%) as a colorless viscous oil.

R_f = 0.32 (silica gel, ethyl acetate/ methanol, 10:2).

^{1}H-NMR, COSY (400 MHz, CD_3OD) δ (ppm) = 5.94 (dddd, ^3J = 17.3 Hz, ^3J = 10.7 Hz, ^3J = 6.0 Hz, ^3J = 5.1 Hz, 1H, Man–O–CH_2–C*H*), 5.30 (*pseudo* dq, ^2J = 17.2 Hz, J = 1.7 Hz, 1H, O–CH_2–CH=C$H_{2,a}$), 5.17 (*pseudo* dq, ^2J = 10.4 Hz, J = 1.5 Hz, 1H, O–CH_2–CH=C$H_{2,b}$), 4.97 (d, ^3J = 1.7 Hz, 1H, H_{Man}-1), 4.22 (*pseudo* ddt, ^2J = 13.1 Hz, ^3J = 5.1 Hz, ^4J = 1.6 Hz, 1H, O–C$H_{2,a}$–CH=CH_2), 4.00 (*pseudo* ddt, ^2J = 13.1 Hz, ^3J = 5.9 Hz, ^4J = 1.4 Hz, 1H, O–C$H_{2,b}$–CH=CH_2), 3.86–3.78 (m, 2H, H_{Man}-2, H_{Man}-6_a), 3.72–3.67 (m, 2H, H_{Man}-3, H_{Man}-6_b), 3.61 (*pseudo* t, ^3J = 9.5 Hz, 1H, H_{Man}-4), 3.53 (ddd, ^3J = 9.8 Hz, ^3J = 5.8 Hz, ^3J = 2.3 Hz, 1H, H_{Man}-5). **^{13}C-NMR, HSQC, HMBC** (100.6 MHz, CD_3OD) δ (ppm) = 135.5 (Man–O–CH_2–*C*H), 117.3 (O–CH_2–CH=*C*H_2), 100.7 (C_{Man}-1), 74.7 (C_{Man}-5), 72.6 (C_{Man}-3), 72.2 (C_{Man}-2), 68.8 (Man–O–*C*H_2–CH), 68.6 (C_{Man}-4), 62.9 (C_{Man}-6).

IR (ATR) λ_{max}/cm^{-1} 3344, 2923, 1453, 1422, 1412, 1261, 1129, 1096, 1053, 1024, 975, 925, 879, 810.

$[\alpha]_D^{24}$ = +62.5° (c = 1.00, H_2O).

ESI-MS: *m/z* (%) = 243.1 (100) [M + Na]$^+$, 244.2 (11) [M + Na]$^+$.

The spectral data are in accordance with literature.[10]

Acetylthiopropyl-α-D-mannopyranoside (3). A solution of allyl α-D-mannopyranose (**2**, 4.24 g, 19.3 mmol, 1 eq.) in thoroughly degassed methanol (100 mL, four freeze-pump-thaw cycles) was treated with thioacetic acid (8.30 g, 7.83 mL, 109 mmol, 6 eq.) and AIBN (40 mg, cat.). The reaction mixture was heated under reflux for 3 days and subsequently the solvent was removed under reduced pressure. The residue was coevaporated with toluene (3 × 60 mL) and purified by flash column chromatography (dichloromethane/ methanol, 10:1.5) to give the title compound (1.83 g, 6.18 mmol, 32%) as a colorless viscous oil. Some of the starting material could be recovered (2.75 g, 12.5 mmol, 65%).

R_f = 0.40 (silica gel, dichloromethane/ methanol, 10:1.5).

^{1}H-NMR, COSY (400 MHz, CD_3OD) δ (ppm) = 4.73 (d, ^3J = 1.7 Hz, 1H, H_{Man}-1), 3.86–3.76 (m, 3H, H_{Man}-2, H_{Man}-6_a, Man–O–C$H_{2,a}$), 3.74–3.67 (m, 2H, H_{Man}-3, H_{Man}-6_b), 3.60 (*pseudo* t, ^3J = 9.5 Hz, 1H, H_{Man}-4), 3.54–3.43 (m, 2H, H_{Man}-5, Man–O–C$H_{2,b}$), 3.03–2.90 (m, 2H, CH_2–SAc), 2.31 (s, 3H, CH_3), 1.89–1.81 (m, 2H, Man–O–CH_2–CH_2). **^{13}C-NMR, HSQC, HMBC** (100.6 MHz, CD_3OD) δ (ppm) = 197.4 (C=O), 101.6 (C_{Man}-1), 74.7 (C_{Man}-5), 72.6 (C_{Man}-3), 72.2 (C_{Man}-2), 68.6 (C_{Man}-4), 66.9 (Man–O–*C*H_2), 62.9 (C_{Man}-6), 30.7 (Man–O–CH_2–*C*H_2), 30.5 (*C*H_3), 26.9 (*C*H_2–SAc).

IR (ATR) λ_{max}/cm^{-1} 3354, 2920, 1688, 1414, 1354, 1321, 1129, 1086, 1051, 1022, 958, 915, 810.

$[\boldsymbol{\alpha}]_{\boldsymbol{D}}^{\boldsymbol{24}}$ = +51.5° (c = 1.00, MeOH).

ESI-MS: *m/z* (%) = 319.1 (100) $[M + Na]^+$, 320.2 (9) $[M + Na]^+$.

HRMS (ESI): Calculated for $[C_{11}H_{20}O_7S + Na]^+$: 319.0827 found: 319.0830.

Representative procedure for the synthesis of thiopropyl-α-D-mannopyranoside (4). Thiopropyl-α-D-mannopyranoside was synthesized freshly prior to use to avoid the formation of disulfide. Acetylthiopropyl-α-D-mannopyranoside (1.0 g, 3.37 mmol, 1 eq) was dissolved in dry methanol and cooled down to 0 °C. Sodium methylate solution 5.4 M (1 mL, 5.06 mmol, 1.5 eq) in dry methanol was added and the solution was stirred for 2 h whereas the solution became turbid. Subsequently, cation exchanger Amberlite® IR-120 was added, stirred for several minutes until the supension became a clear solution. The cation exchanger was filtered and the solvent was removed under reduced pressure. Thiopropyl-α-D-mannopyranoside was obtained as colorless viscous oil in quantitative yield (860 mg, 3,37 mmol).

^{1}H NMR (250 MHz, Deuterium Oxide) δ 3.91 – 3.34 (m, 9H), 2.49 (t, *J* = 7.1 Hz, 2H), 1.95 – 1.62 (m, *J* = 7.5 Hz, 2H). **^{13}C NMR** (75 MHz, Deuterium Oxide) δ 99.71, 72.78, 70.58, 70.04, 66.74, 65.80, 60.91, 48.86, 32.49, 20.49.

2.2 Surfactant synthesis

Synthesis of Phos-S(1). All Schlenk-tubes were flame-dried prior to use. 1-Octadecanol was used as initiator and 1,8-diazabicyclo[5.4.0]undec-7-ene (DBU) / 1-(3,5-bis(trifluoromethyl)phenyl)-3-cyclohexylthiourea (TU) as catalyst/co-catalyst system. TU (273 mg, 739 µmol, 4.4 eq) and 1-octadecanol were introduced into flame-dried Schlenk-tubes, dissolved in benzene, and dried by lyophilization. An initiator solution in dry DCM was prepared (40 mg mL^{-1}). MEP (924 mg, 6.65 mmol, 40 eq), initiator (45 mg, 166 µmol, 1 eq) and 3 mL of dry DCM were added to the TU co-catalyst. The reaction mixture was cooled down to 5 °C and the polymerization was started by rapid addition of DBU (112 mg, 739 µmol, 4.4 eq). After initiation, the reaction mixture was cooled down to 0°C. The polymerization was quenched after 1.5 h with an excess of acetic acid in DCM. The polymer was precipitated from diethylether, dissolved in water and dialyzed against water over night. After lyophilization the polymer was obtained as colorless viscous liquid (876 mg, 90%).

^{1}**H NMR** (250 MHz, Chloroform-*d*) δ 4.46 – 4.10 (m, 137H), 3.80 (d, *J* = 11.3 Hz, 108H), 1.65 (m, 2H,), 1.24 (s, 30H), 0.96 – 0.74 (m, 3H).

Synthesis of Phos-S(2). All Schlenk-tubes were flame-dried prior to use. 1-octadecanol was used as initiator and DBU/TU as catalyst. TU (273mg, 739 µmol, 5 eq) and 1-octadecanol were introduced into separate flame-dried Schlenk-tubes, dissolved in dry benzene and dried by lyophilization. MEP (416 mg, 3.02 mmol, 20 eq) was added to the TU co-catalyst. A stock solution of the initiator in dry DCM was prepared at a concentration of 40 mg mL^{-1}. 1.02 mL (40.8 mg, 151 µmol, 1 eq) of initiator stock solution and 2 mL of dry DCM were added to the MEP/TU mixture. The reaction mixture was cooled down to 5 °C and the polymerization was started by rapid addition of DBU. Subsequently, the temperature was adjusted to 0 °C. The reaction was stirred for 1.5 h at 0 °C. After 1.5 h 0.1 mL of the reaction mixture was withdrawn for SEC analysis. 1.07 g OPEMA (4.52 mmol, 30 eq) dissolved in 3 mL of dry DCM were added and the reaction was stirred further for 1 h. The polymerization was quenched by rapid addition of an excess of acetic acid dissolved in DCM (20 mg mL^{-1}). The ice bath was removed and the mixture was stirred for 5 minutes at ambient temperature. The polymer was obtained in quantitative yield as colorless viscous oil by precipitation from diethyl ether, subsequent centrifugation and decantation.

^{1}**H NMR** (250 MHz, Chloroform-*d*) δ 6.14 (s, 19H), 5.60 (s, 19H), 4.54 – 4.12 (m, 224H), 3.80 (dd, *J* = 11.2, 5.0 Hz, 60H), 1.94 (s, 59H), 0.91 – 0.81 (t, *J* = 6.2 Hz, 3H). ^{31}**P NMR** (121 MHz, Methylene Chloride-d_2) δ -0.16, -1.27.

Synthesis of Phos-S(3). All Schlenk-tubes were flame-dried prior to use. 1-Octadecanol was used as initiator and DBU / TU as catalyst/co-catalyst system. TU (205 mg, 554 µmol, 5.7 eq) and 1-octadecanol were introduced into separate flame-dried Schlenk-tubes, dissolved in dry benzene and dried by lyophilization. OPEMA (920 mg, 3.89 mmol, 40 eq) was added to the co-catalyst. The initiator stock solution was prepared at a concentration of 50 mg mL^{-1}. 0.53 mL (26.3 mg, 97 µmol, 1 eq) of the initiator stock solution and 4 mL of dry DCM were added to the TU/OPEMA mixture and cooled down to 0 °C. The reaction was started by rapid addition of DBU (84 mg, 554 µmol, 5.7 eq) to the reaction mixture. The reaction was stirred for 1.5 h at 0 °C and quenched by rapid addition of an excess of acetic acid dissolved in DCM. The polymer was obtained as colorless viscous liquid (918 mg, 96%) after precipitation from diethylether and subsequent decantation.

^{1}**H NMR** (250 MHz, Chloroform-*d*) δ 6.14 (s, 37H), 5.60 (s, 37H), 4.67 – 4.17 (m, 285 H), 1.94 (s, 112H), 1.18 (s, 30H), 0.87 (t, *J* = 6.2 Hz, 3H). ^{31}**P NMR** (121 MHz, Chloroform-*d*) δ -1.30.

Table S1. Surfactant characterization without mannose units

	n(MEP)	n (OPEMA)	M_n / g mol^{-1a}	$Đ^b$	γ / mN^c
**Phos-S(1)**	36	-	5,200	1.33	47.60 ± 0.05
**Phos-S(2)**	20	19	7,500	1.50	-
**Phos-S(3)**	-	37	9,000	1.23	-

[a] determined by ^{1}H NMR spectroscopy; [b] determined by SEC in DMF; [c] determined by ring tensiometry at a surfactant concentration of 1 gL^{-1} in distilled water.

Representative procedure for the post-polymerization modification of polyphosphoester surfactants Phos-S2 and Phos-S3. Phos-S3 (100 mg, 19 µmol) was dissolved in 50 mL methanol and thiopropyl-α-D-mannopyranoside (495 mg, 1.9 mmol, (5 eq per methacrylate functionality)) was added. DMAP (40 mg, 0.3 mmol)) was added as catalyst and tris (2-carboxyethyl) phosphine (TCEP) (20 mg, 0.07 mmol) to reduce the formed disulfide. The was conducted at ambient temperature. The progress of the reaction was monitored by ^{1}H NMR spectroscopy. When the signals of the double bond in the ^{1}H NMR spectrum have disappeared the reaction was completed and methanol was removed under reduced pressure and the residue was dissolved in water and charged into a dialysis tube (MWCO = 3,500 kDa). The crude product was dialyzed against water until DMAP, TCEP and the excess of thiopropyl-α-D-mannopyranoside were removed. After lyophilization, the product was obtained as white solid (105 mg, 42 %).

NMR-Data for Phos-S2-Man^{1}H NMR (500 MHz, Deuterium Oxide) δ 4.42 – 4.19, 3.95 – 3.44 (m), 2.86 – 2.52 (m), 1.82 (m), 1.20 (m), 0.93 (m). **^{13}C NMR** (125 MHz, Deuterium Oxide) δ 99.78, 72.75, 70.69, 70.14, 67.10, 66.52, 66.04, 66.01, 63.58, 60.85, 55. 25, 40.22, 34.52, 29.87, 28.63, 16.32. **^{31}P NMR** (202 MHz, Deuterium Oxide) δ 1.48, -0.10, -1.43

NMR-Data for Phos-S3-Man^{1}H NMR (500 MHz, Deuterium Oxide) δ 4.42 – 4.19 (m), 3.95 – 3.44 (m), 2.76 (m), 2.68 (m), 2.63 – 2.53 (m), 1.82 (m), 1.20 (m), 0.91 (m). **^{13}C NMR** (125 MHz, D_2O) δ 99.77, 72.74, 70.69, 70.13, 67.09, 66.03, 60.85, 40.22, 34.70, 28.78, 28.71, 16.36. **^{31}P NMR** (202 MHz, Deuterium Oxide) δ 0.33, -1.42.

Table S2. Surfactant characterization without mannose units

	n(Man)[a]	M_n / g mol^{-1}[a]	γ / mN[b]
**Phos-S(2)-Man**	19	12,300	47.34 ± 0.07
**Phos-S(3)-Man**	37	18,400	51.21 ± 0.03

[a] determined by ^{1}H NMR spectroscopy; [b] determined by ring tensiometry at a surfactant concentration of 1 gL^{-1} in distilled water.

2.3 Nanoparticle Preparation

Synthesis of PS/PMMA model nanocarriers. A macroemulsion was prepared with a continuous phase containing sodium dodecyl sulfate (SDS) (60 mg, 0.21 mmol) as surfactant in 24 g MilliQ water and a dispersed phase containing stabilizer-free styrene (6 g, 57.6 mol), hexadecane (250 mg, 0.9 mmol) as ultrahydrophobe, Bodipy methacrylate (6 mg, 1.3·10^{-5} mol) as fluorescent dye and azoisobutyronitrile (AIBN) (100 mg, 0.6 mmol) as oil soluble initiator. For the synthesis of PMMA particles styrene was replaced by methyl methacrylate (MMA) (6 g, 59.9 mmol) as monomer, while all other components remained the same. In both cases, both phases were homogenized by mechanical stirring and subsequently, the organic phase was added slowly to the stirring aqueous phase. The macroemulsion was stirred for 1h at highest speed. Subsequently, the macroemulsion was ultrasonicated with a Branson Sonifier (1/2“ tip, 6.5 nm diameter) for 3 min (program: 10 s puls and 3 s pause) at 70% amplitude under ice cooling to obtain a miniemulsion. The miniemulsion was directly transferred into a 50 mL flask, heated to 72°C and stirred overnight.

Scheme S1: Reaction scheme for the radical polymerization:

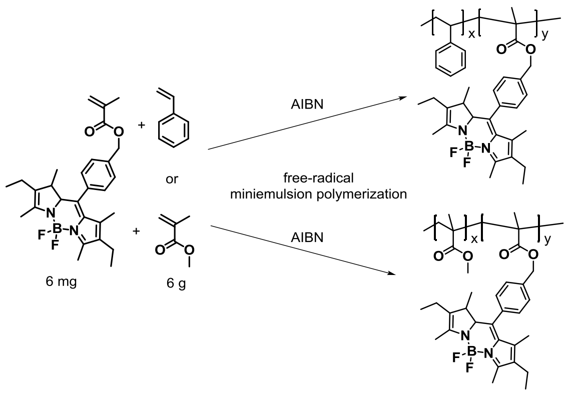

Loading of nanocarriers with surfactants. The nanocarrier dispersion (2 %, 100 µL) was washed with water (1 mL), centrifuged (20 000 g, 1 h) and resuspended in the respective surfactant solution (1%, 1 mL).

2.4 NMR Spectra

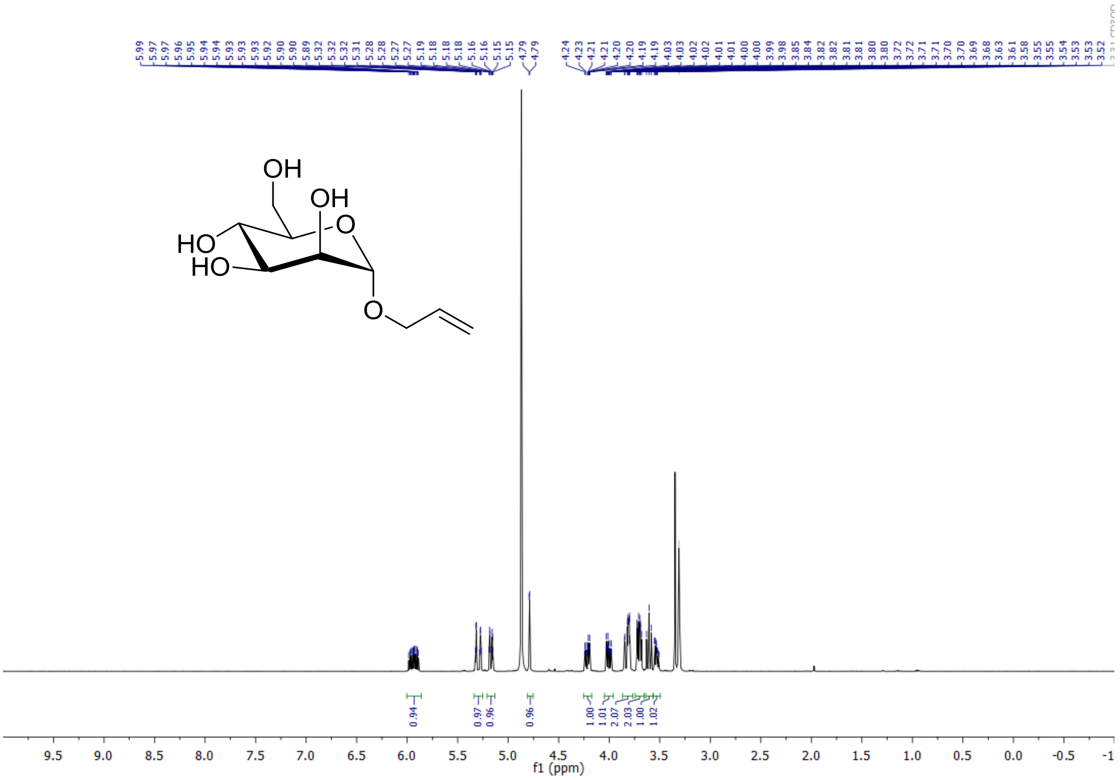

Figure S1. ^{1}H NMR (400 MHz, CD_3OD), compound **2**

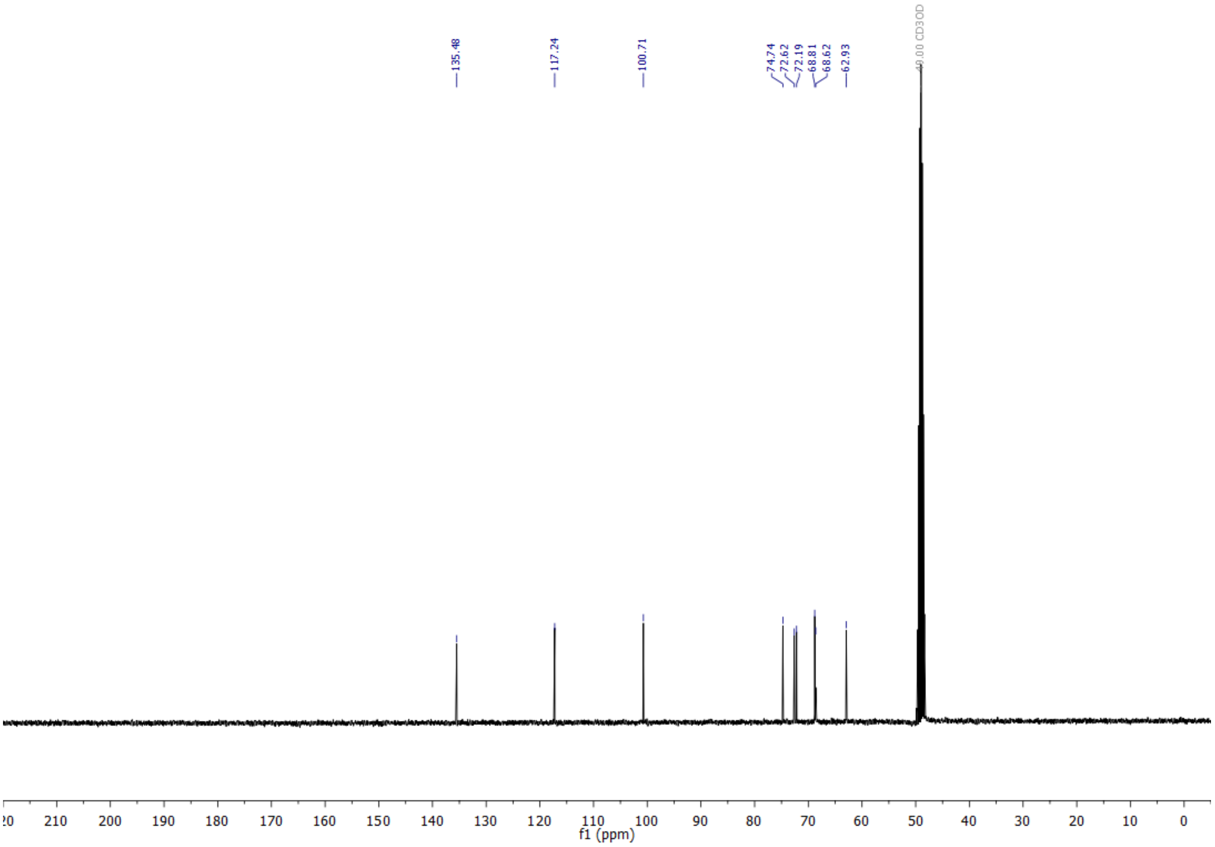

Figure S2. ^{13}C NMR (100.6 MHz, CD_3OD), compound **2**

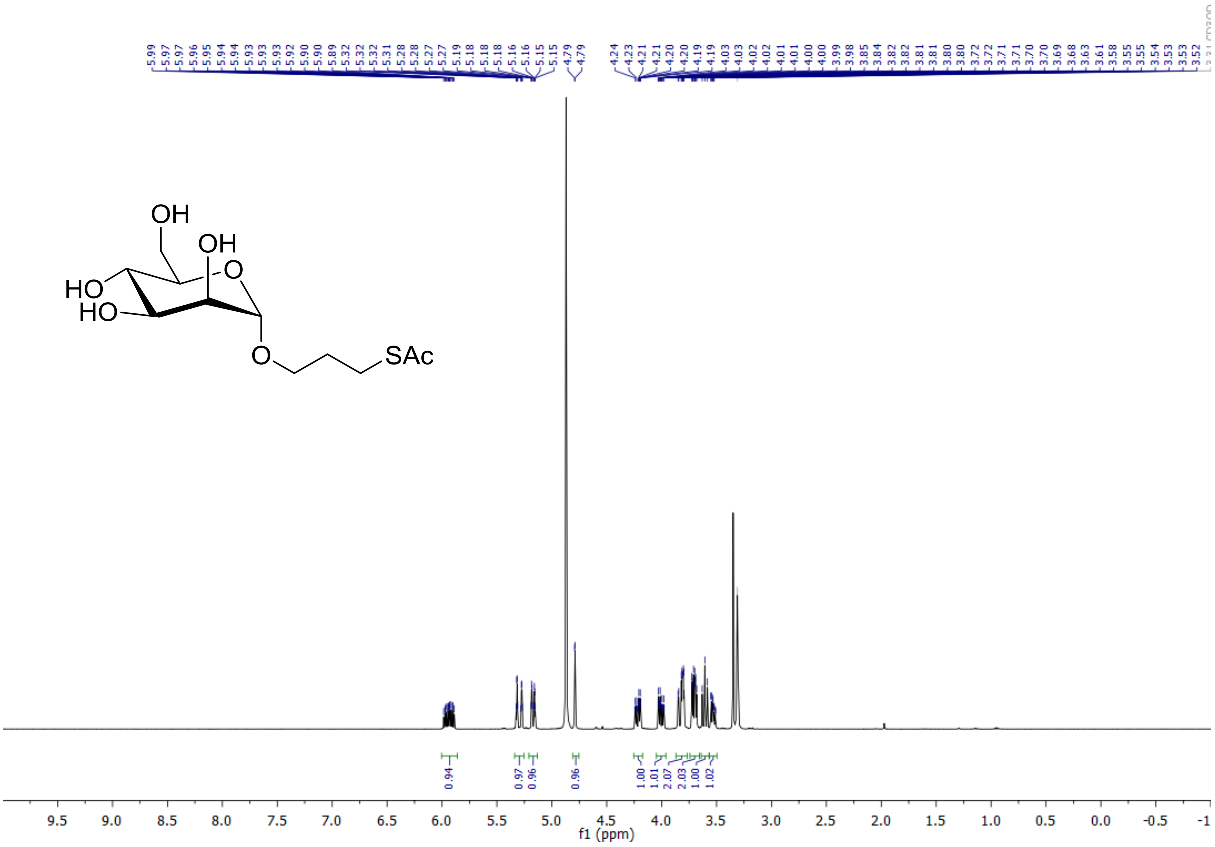

Figure S3. ^{1}H NMR (400 MHz, CD_3OD), compound **3**

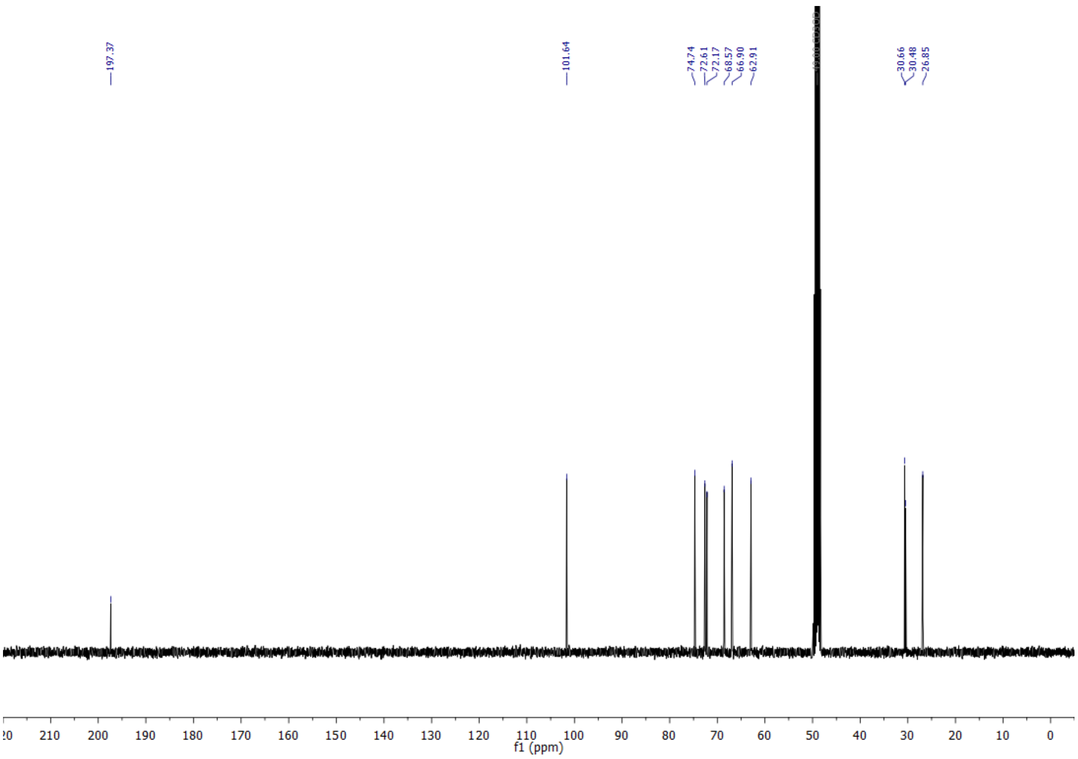

Figure S4. ^{13}C NMR (100.6 MHz, CD_3OD), compound **3**

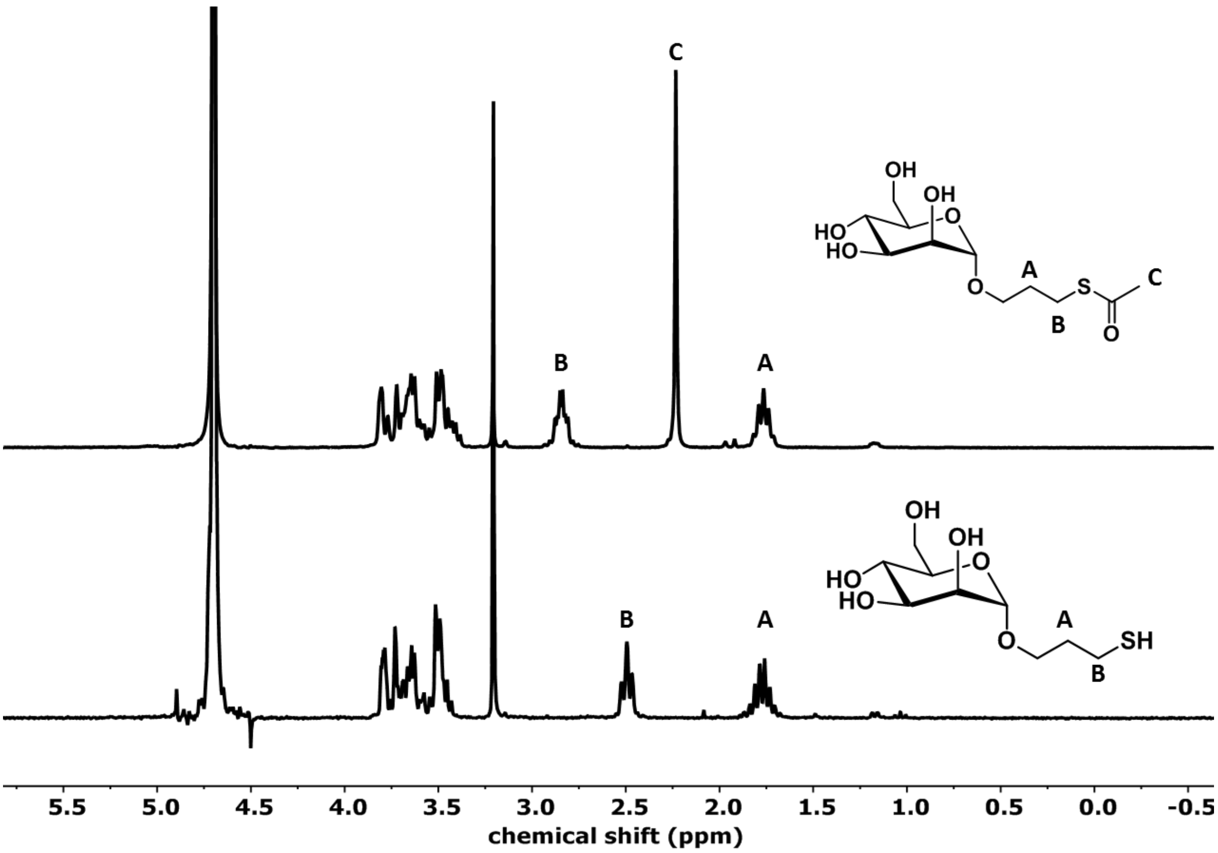

Figure S5. ^{1}H NMR of compound (3) (top) and compound (4) (bottom) (250 MHz, D_2O, 290K).

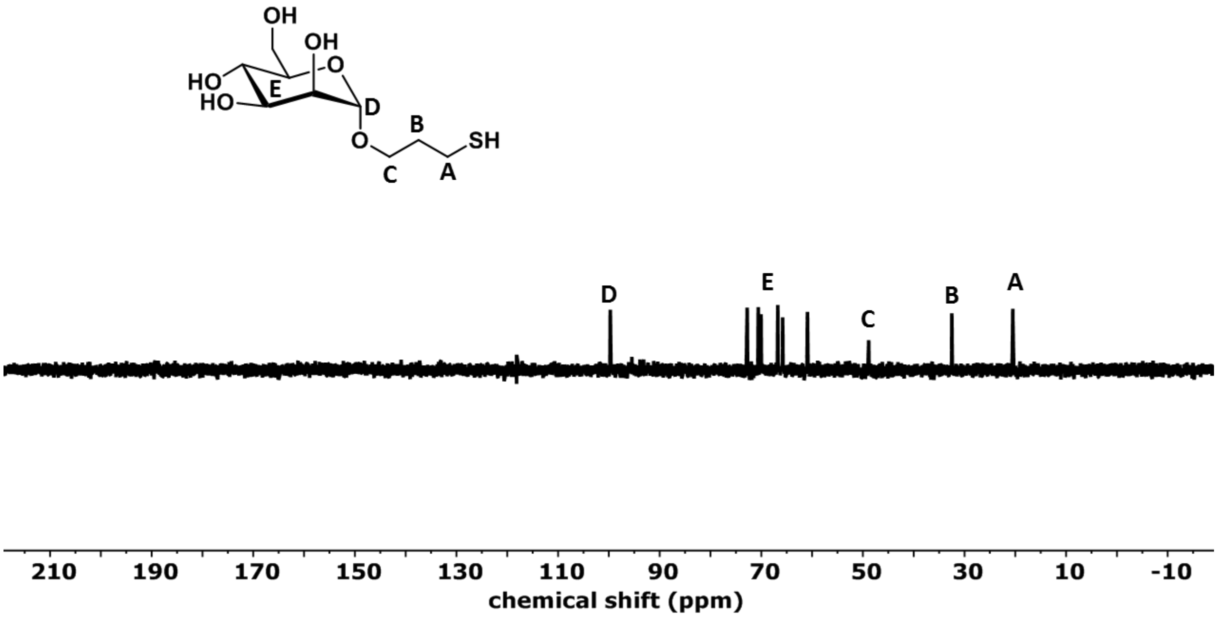

Figure S6. ^{13}C NMR of (4) (75 MHz, D_2O, 300K)

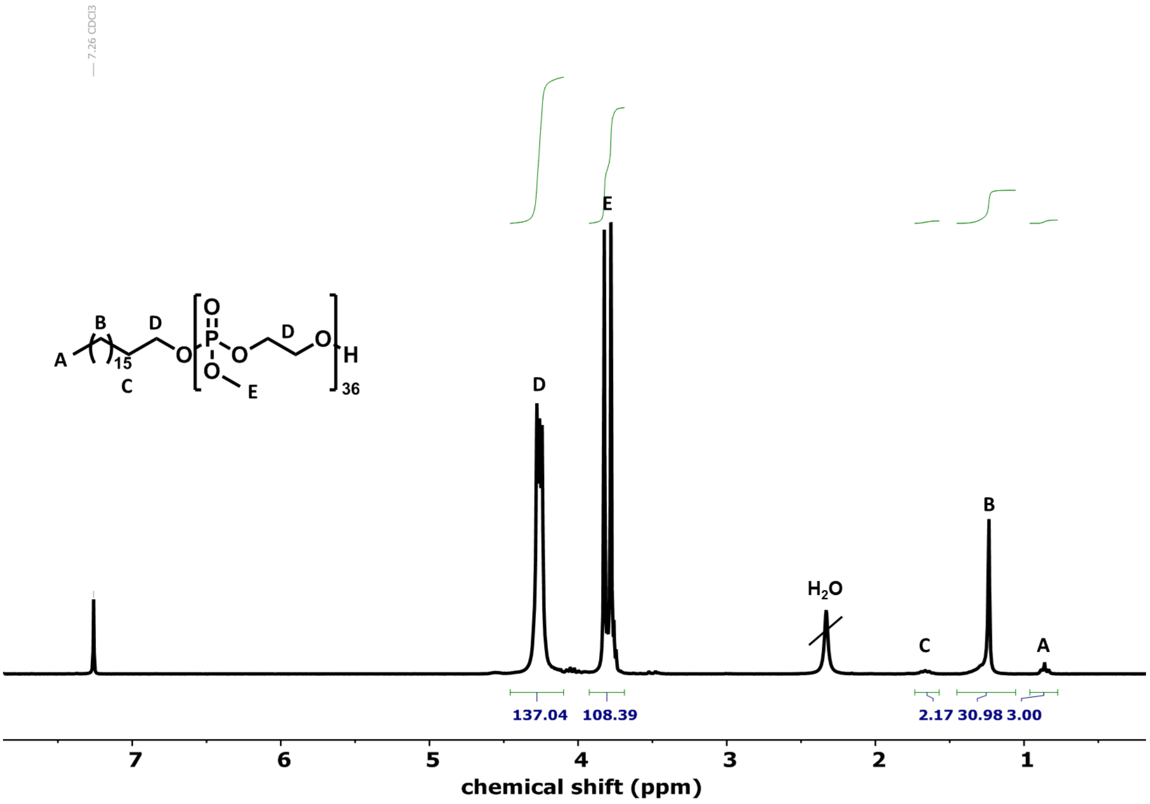

Figure S7. ^{1}H NMR of Phos-S1 (250 MHz, $CDCl_3$, 290 K).

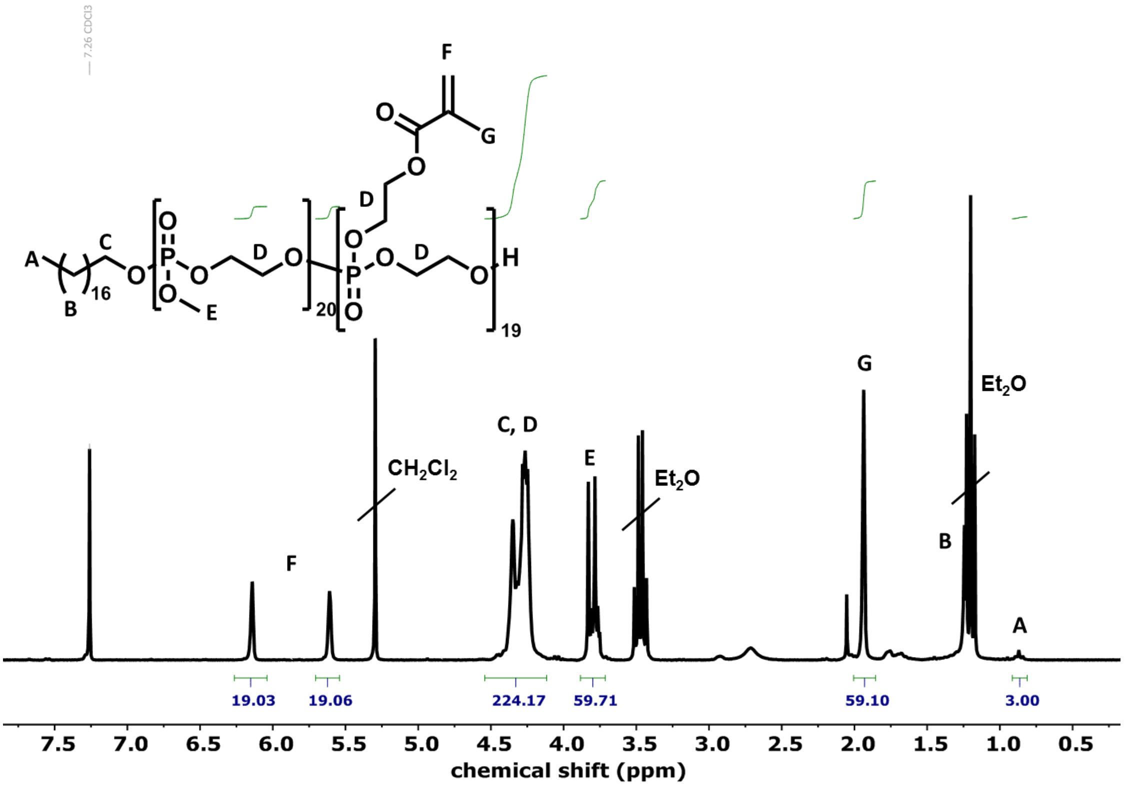

Figure S8. ^{1}H NMR of Phos-S2 (250 MHz, $CDCl_3$, 290 K).

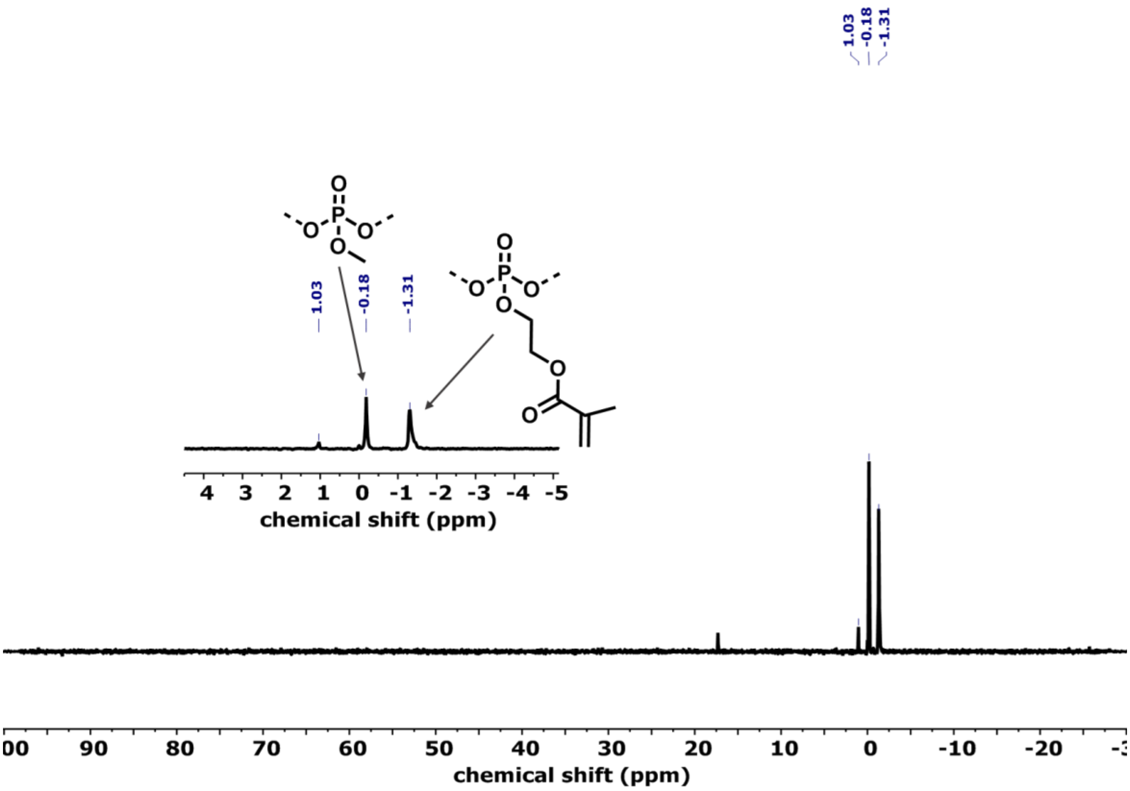

Figure S9. ^{31}P NMR of Phos-S2 (121 MHz, CD_2Cl_2, 300 K).

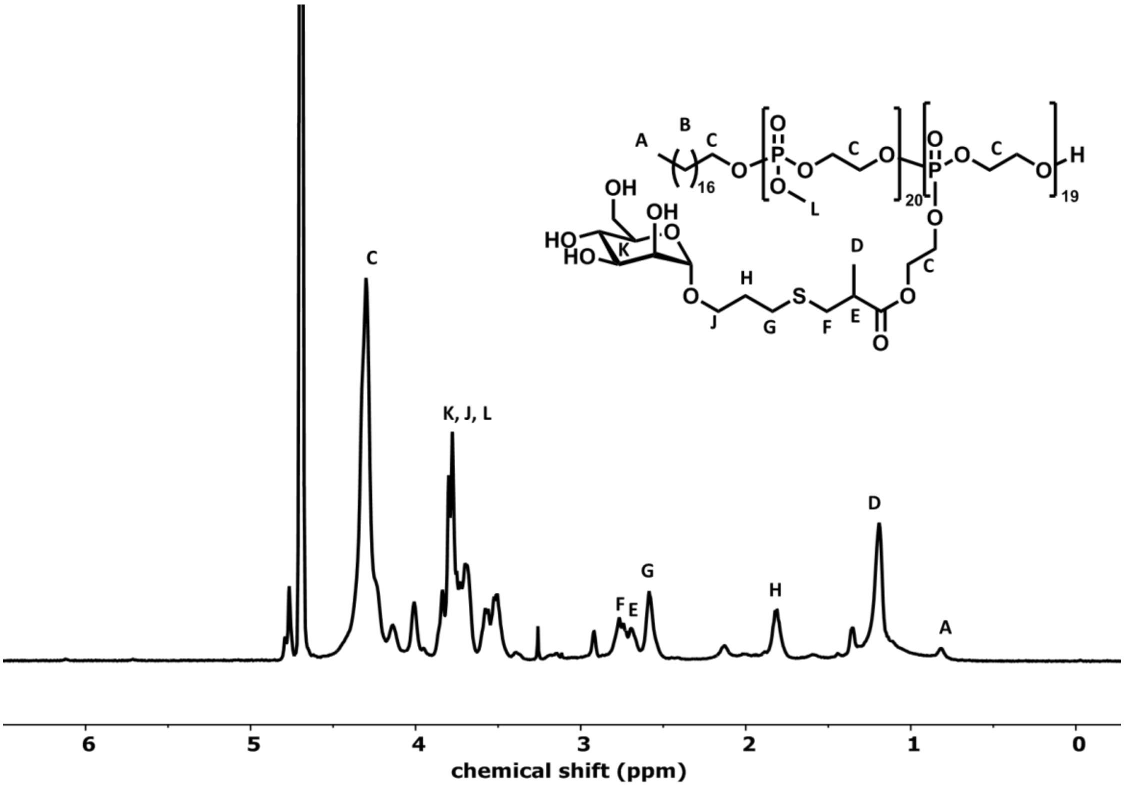

Figure S10. ^{1}H NMR Phos-S2-Man (500 MHz, D_2O, 298K)

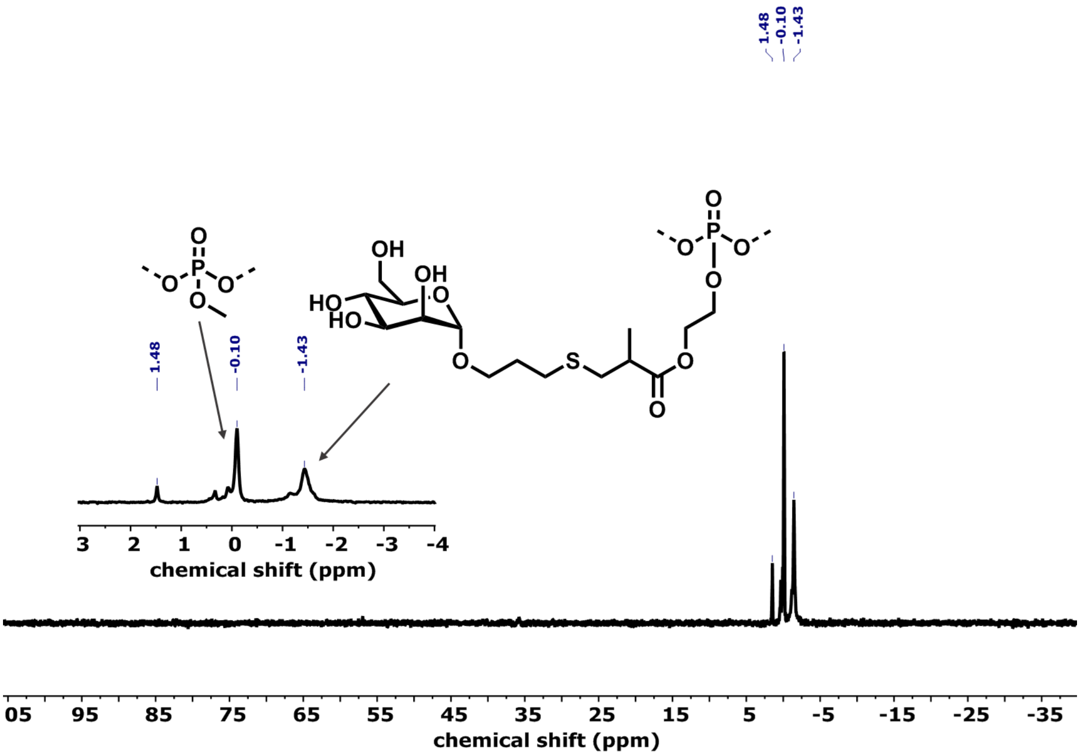

Figure S11. ^{31}P NMR Phos-S2-Man (202 MHz, D_2O, 298K)

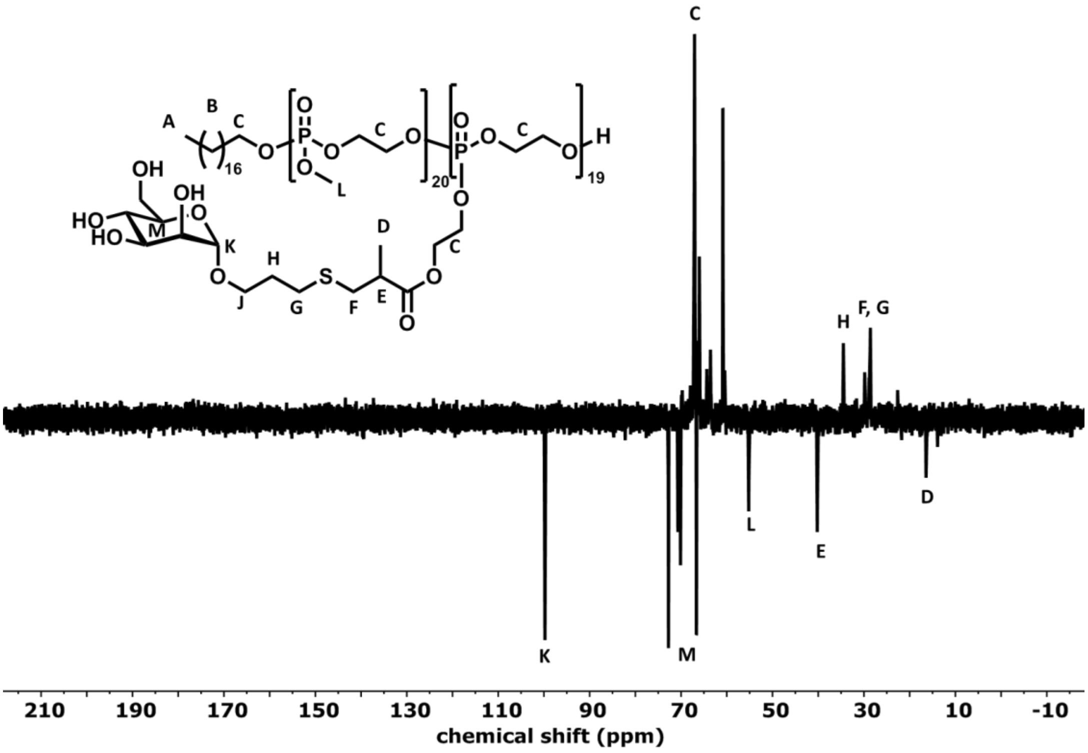

Figure S12. ^{13}C NMR (JMOD) of Phos-S2-Man (125 MHz, D_2O, 298K).

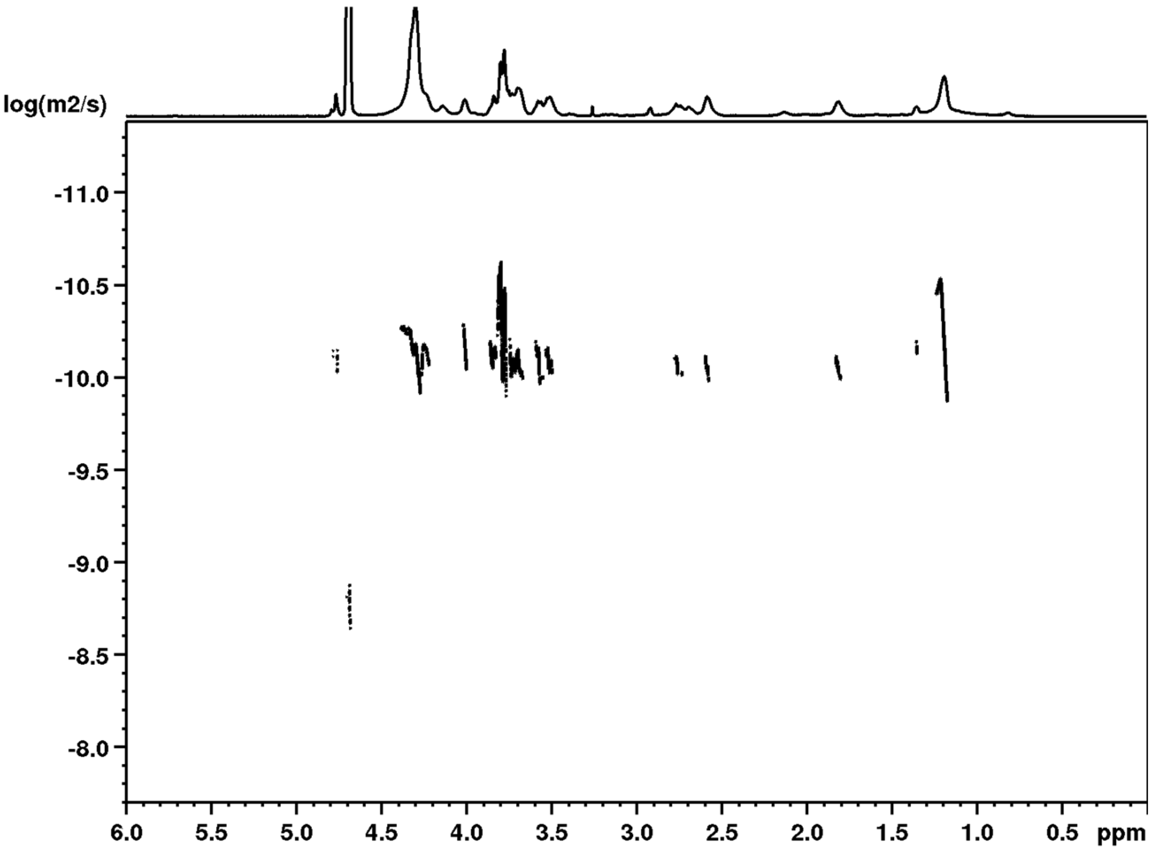

Figure S13. ^{1}H DOSY NMR Phos-S2-Man (500 MHz, D_2O, 298K)

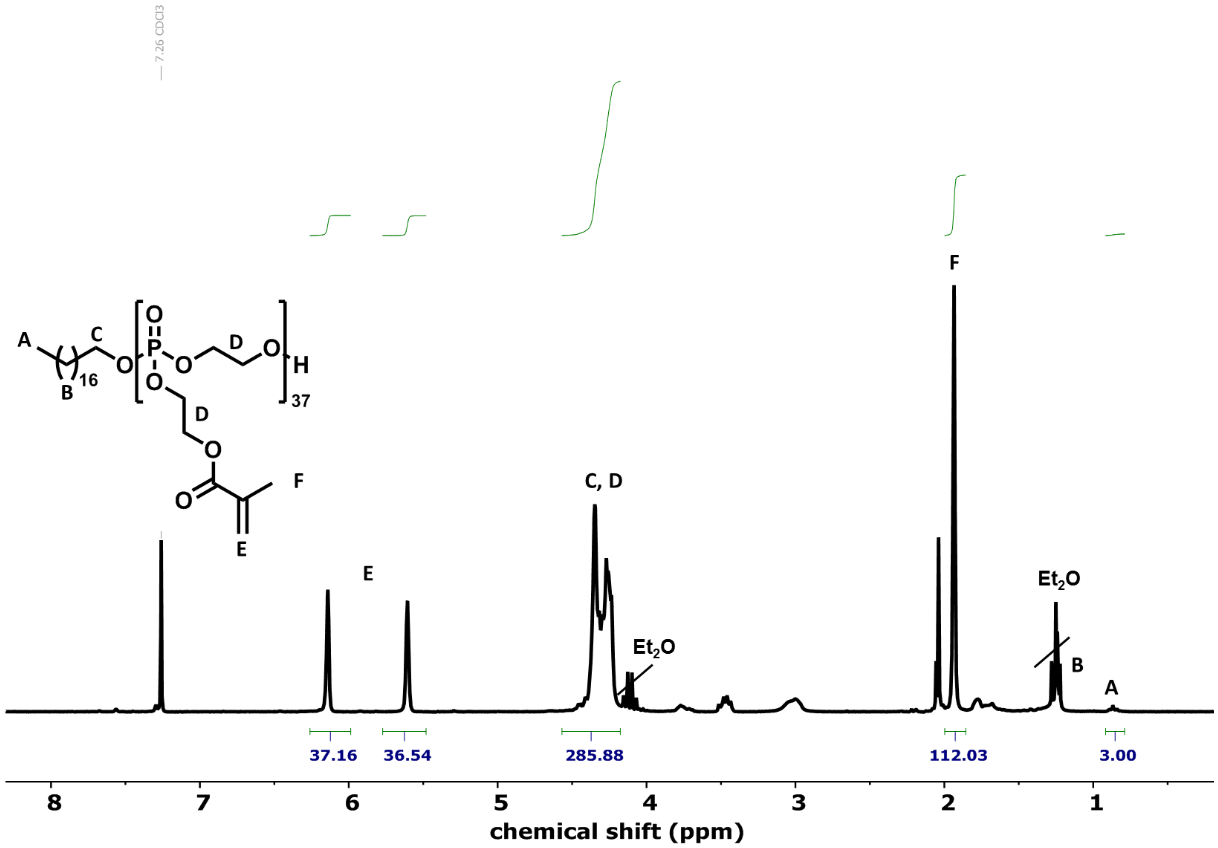

Figure S14. ^{1}H NMR of Phos-S3 (250 MHz, $CDCl_3$, 290 K).

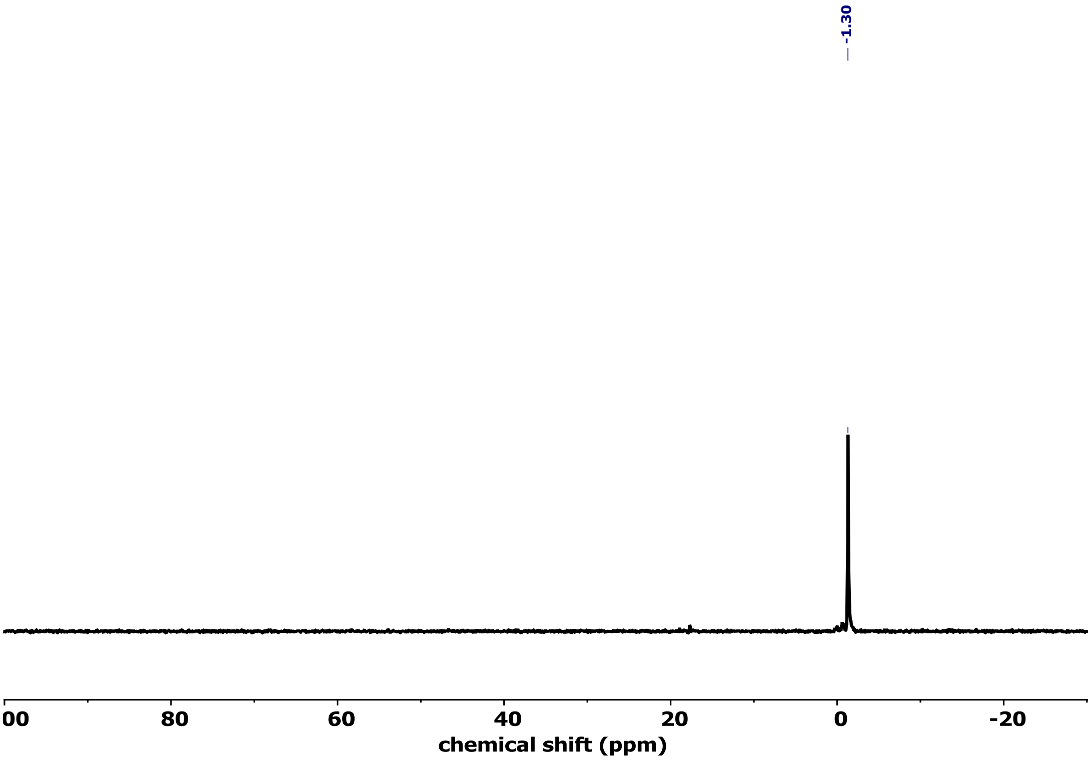

Figure S15. ^{31}P NMR of Phos-S3 (121 MHz, $CDCl_3$, 300 K).

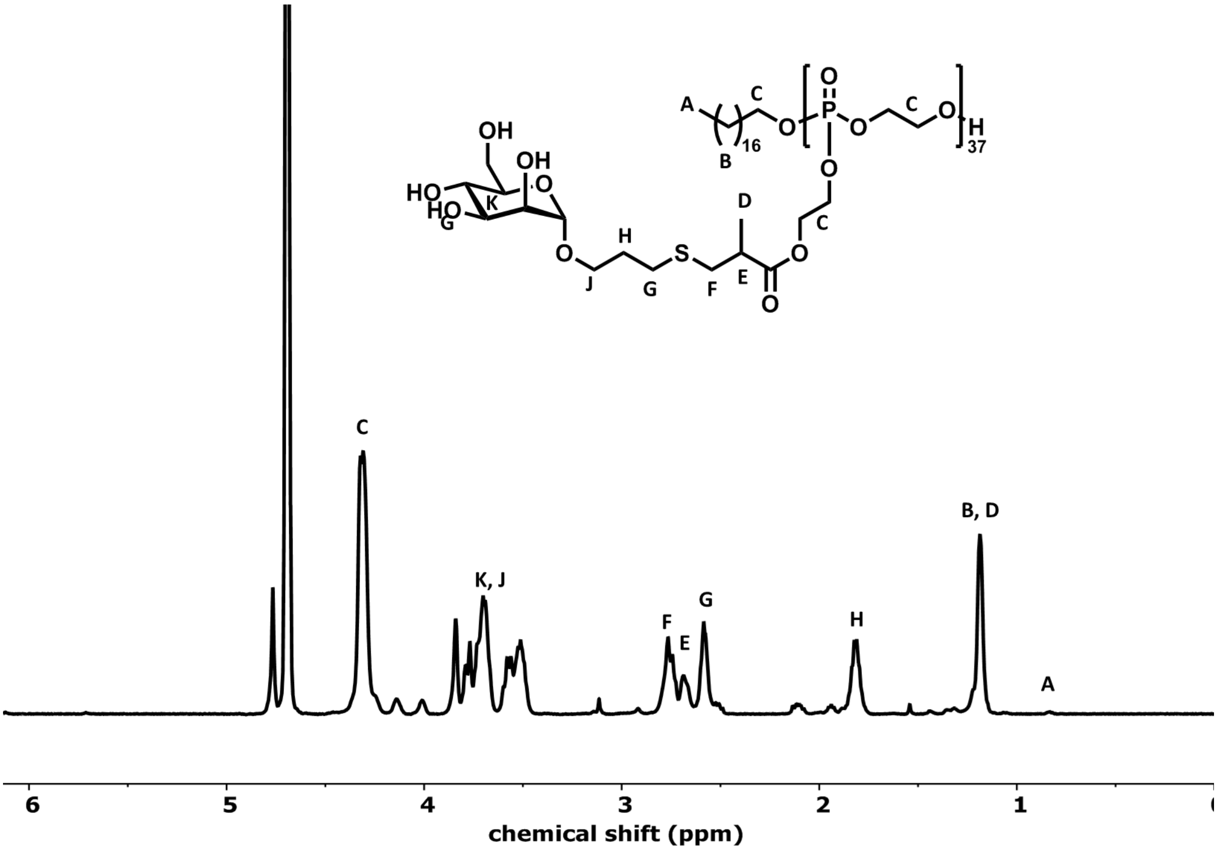

Figure S16. ^{1}H NMR Phos-S3-Man (500 MHz, D_2O, 298K).

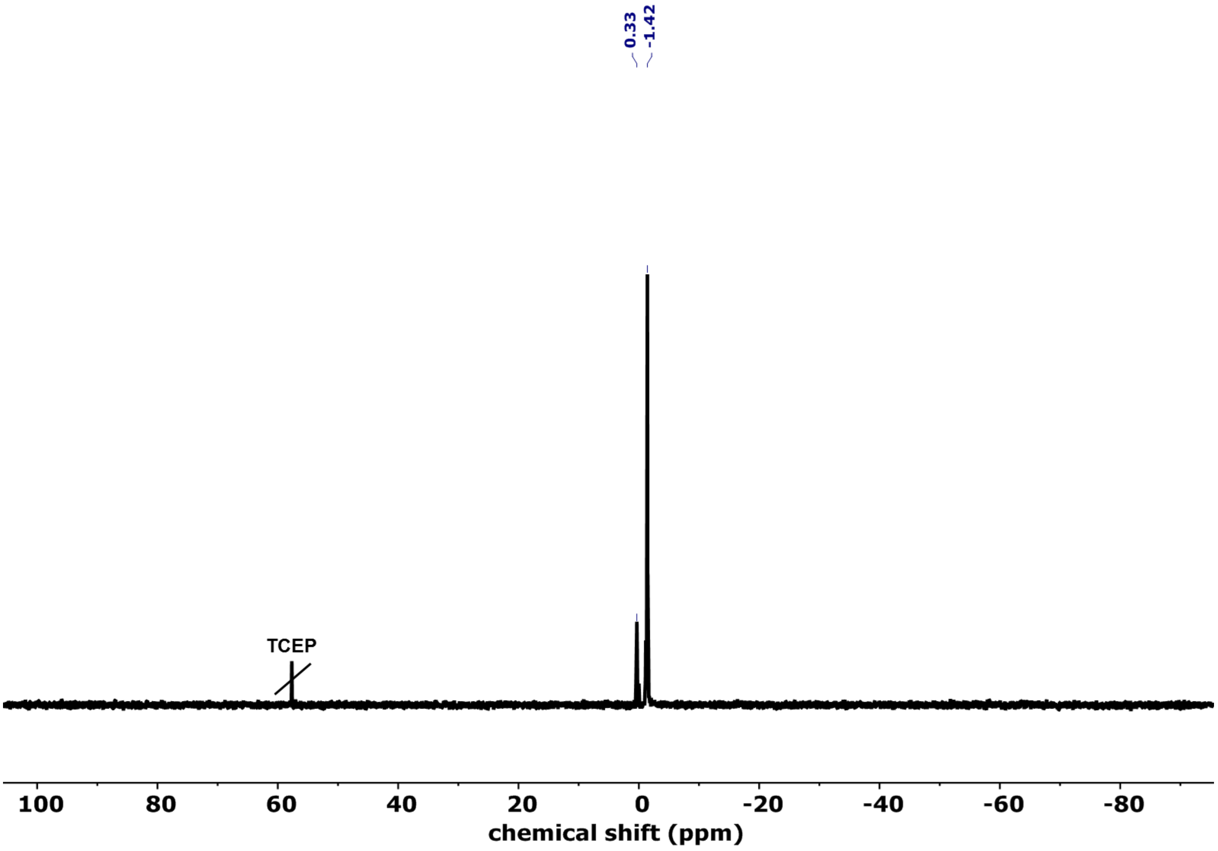

Figure S17. ^{31}P NMR Phos-S3-Man (202 MHz, D_2O, 298K)

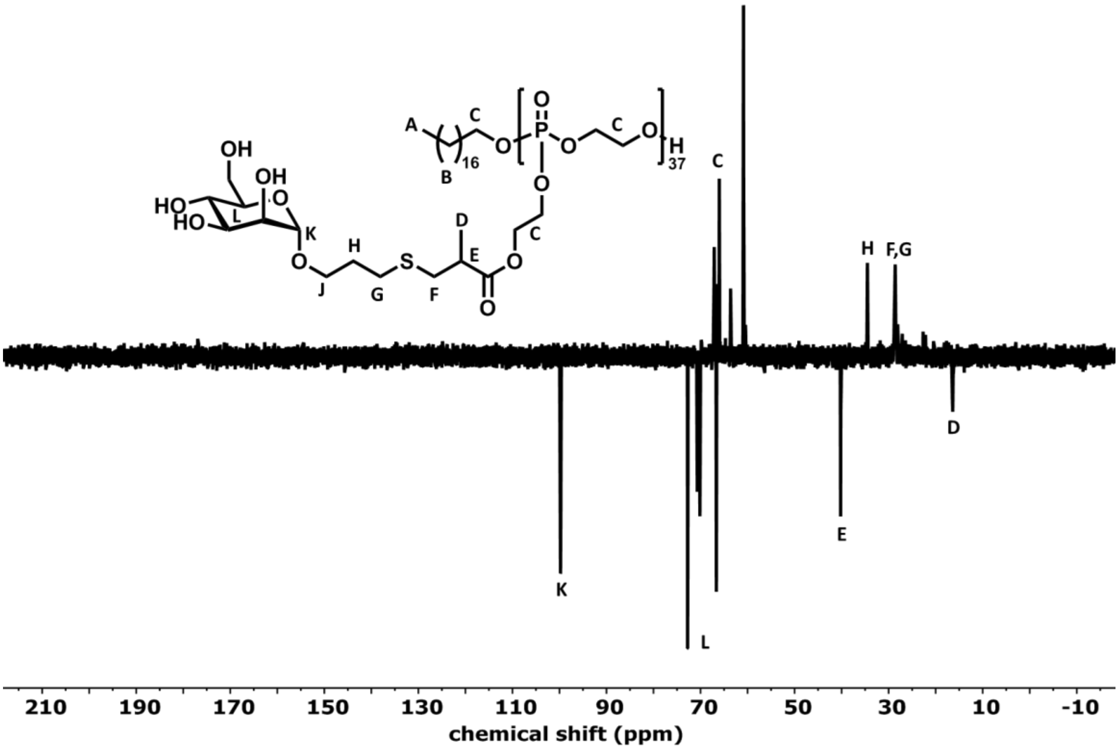

Figure S18. ^{13}C NMR (JMOD) of Phos-S3-Man (125 MHz, D_2O, 298K).

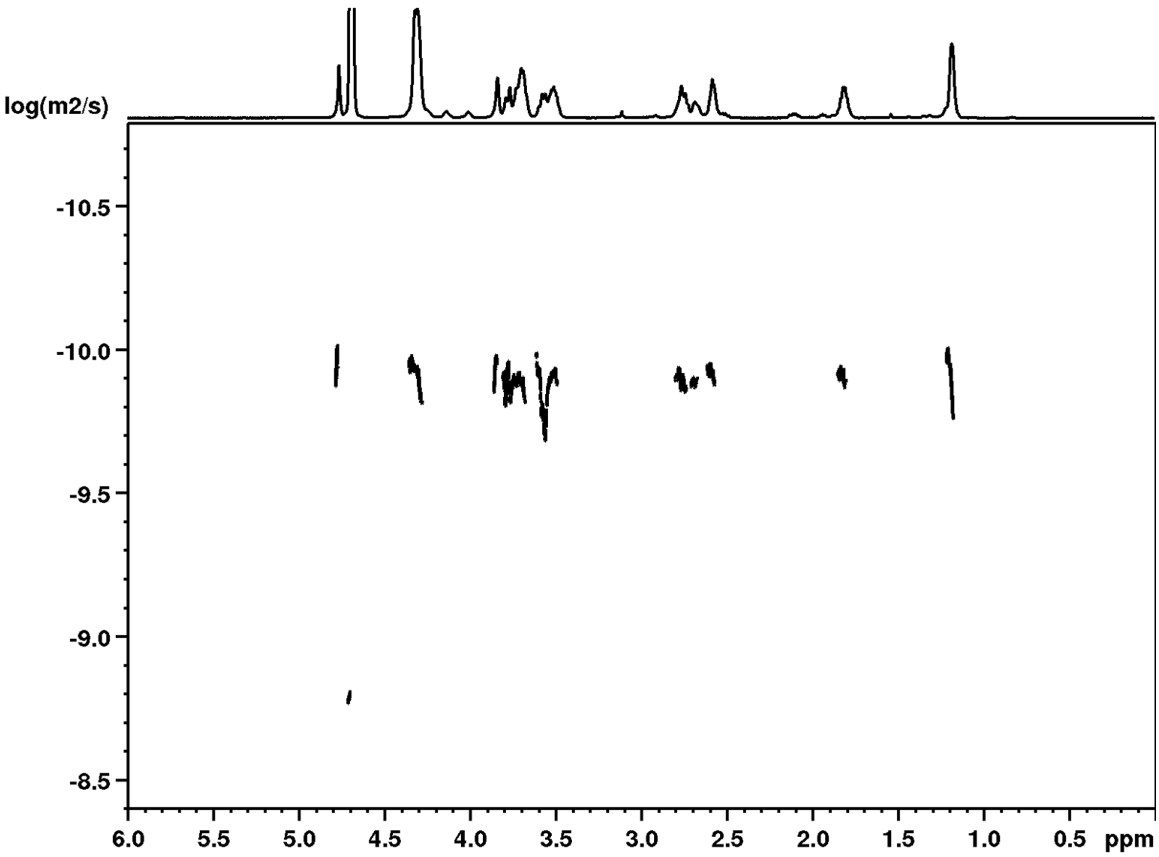

Figure S19. ^{1}H DOSY NMR Phos-S3-Man (500 MHz, D_2O, 298K)

2.5 SEC

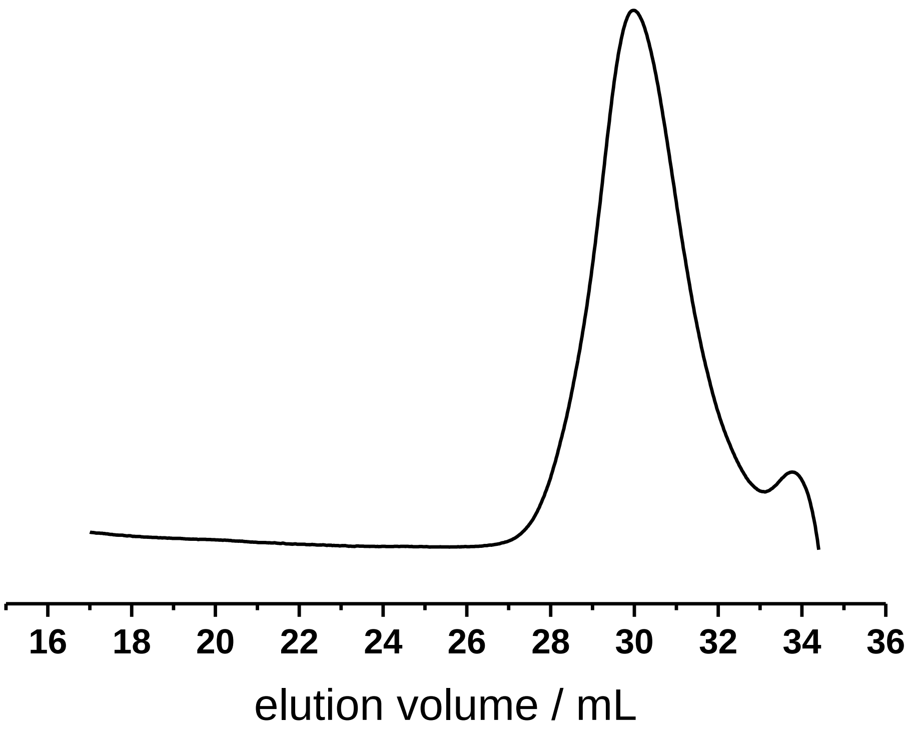

Figure S20. SEC elugram of Phos-S1in DMF at 60°C.

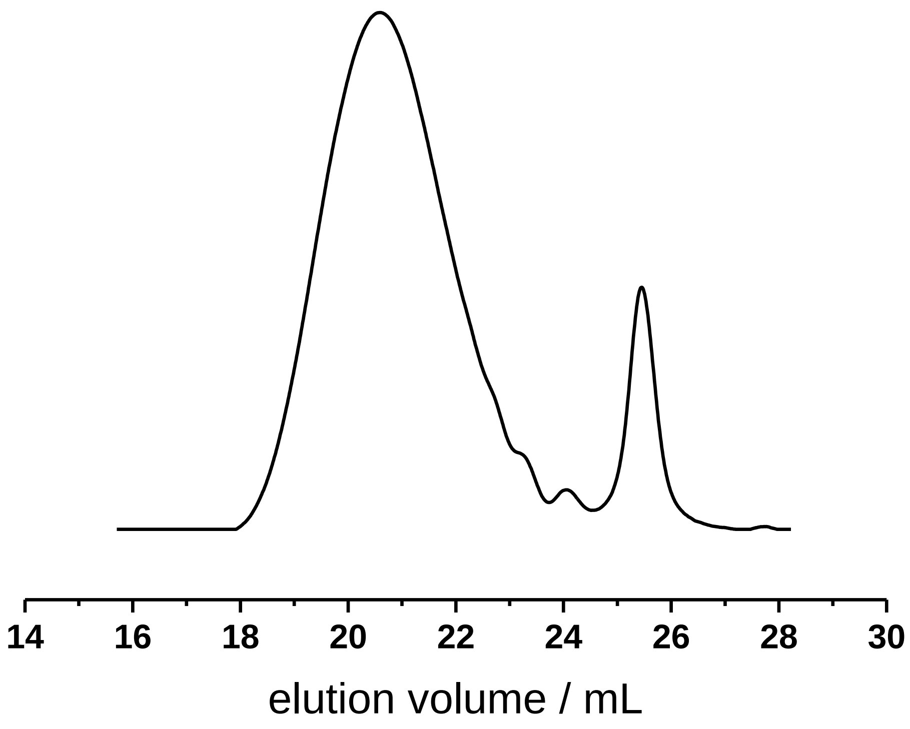

Figure S21. SEC elugram of Phos-S3 in DMF at 50°C.

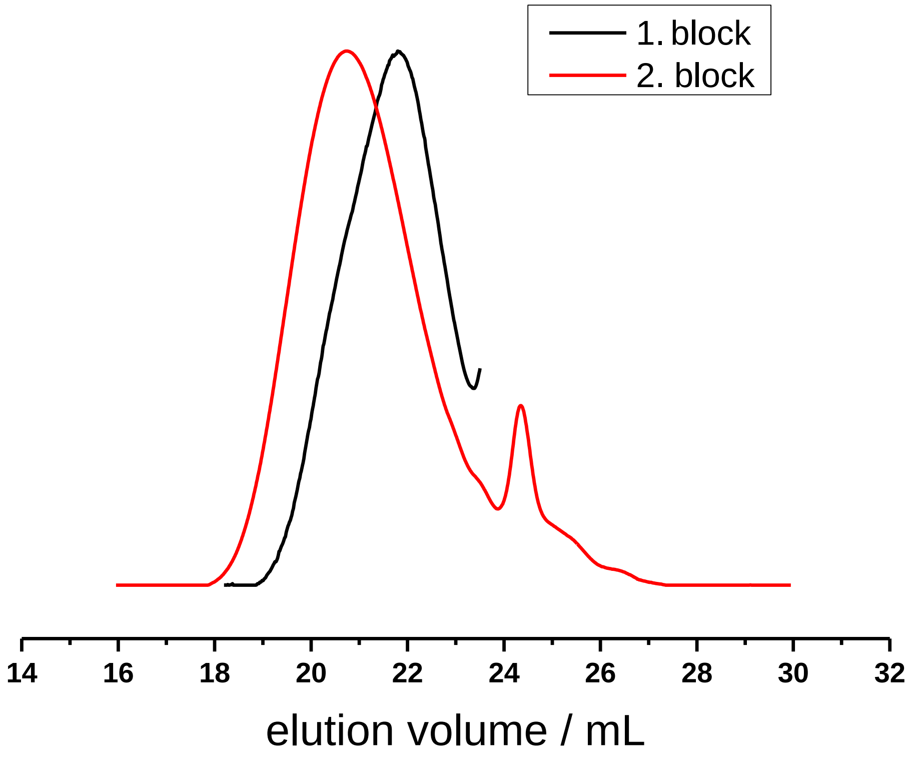

Figure S22. SEC elugram of Phos-S2 in DMF at 50°C.

3. Colloid stability and toxicity

Table S3. Physico-chemical properties of the polymeric nanocarries without surfactant coating.

	Name	Ø / nm	ζ / mV
**Polystyrene**	PS	101 ± 12	-52 ± 13
**Poly (methyl methacrylate)**	PMMA	114 ± 15	-46 ± 12

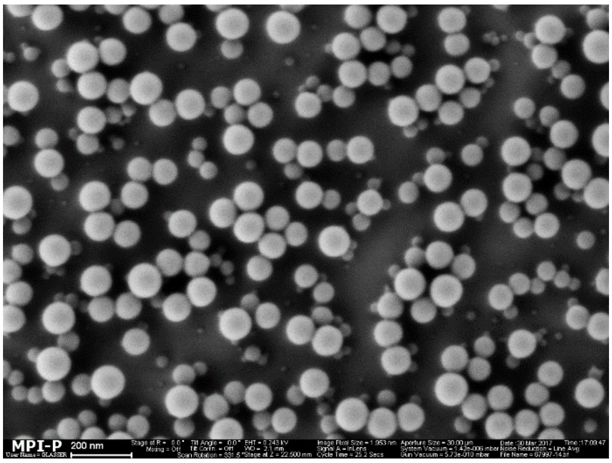

Figure S23. SEM picture of polystyrene nanoparticles.

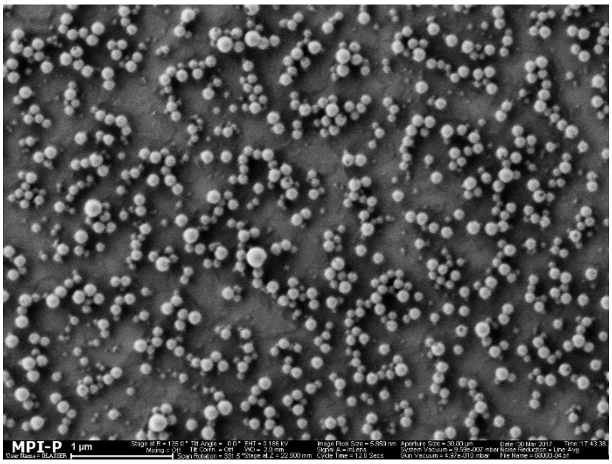

Figure S24. SEM picture of poly (methyl methacrylate) nanoparticles.

a)

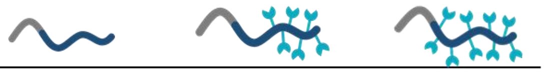

D_h / nm	Phos-S(1)	Phos-S(2)-Man	Phos-S(3)-Man
**PMMA**	159 ± 53	144 ± 58	152 ± 49
**PS**	123 ± 27	122 ± 30	127 ± 31

b)

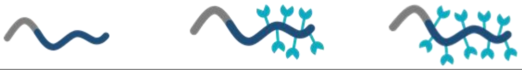

D_h / nm	Phos-S(1)	Phos-S(2)-Man	Phos-S(3)-Man
**PMMA**	156 ± 41	157 ± 49	170 ± 58
**PS**	138 ± 29	152 ± 39	151 ± 40

Figure S25. Physicochemical properties of the polymeric nanocarries with surfactant coating before (a) and after plasma incubation (b).

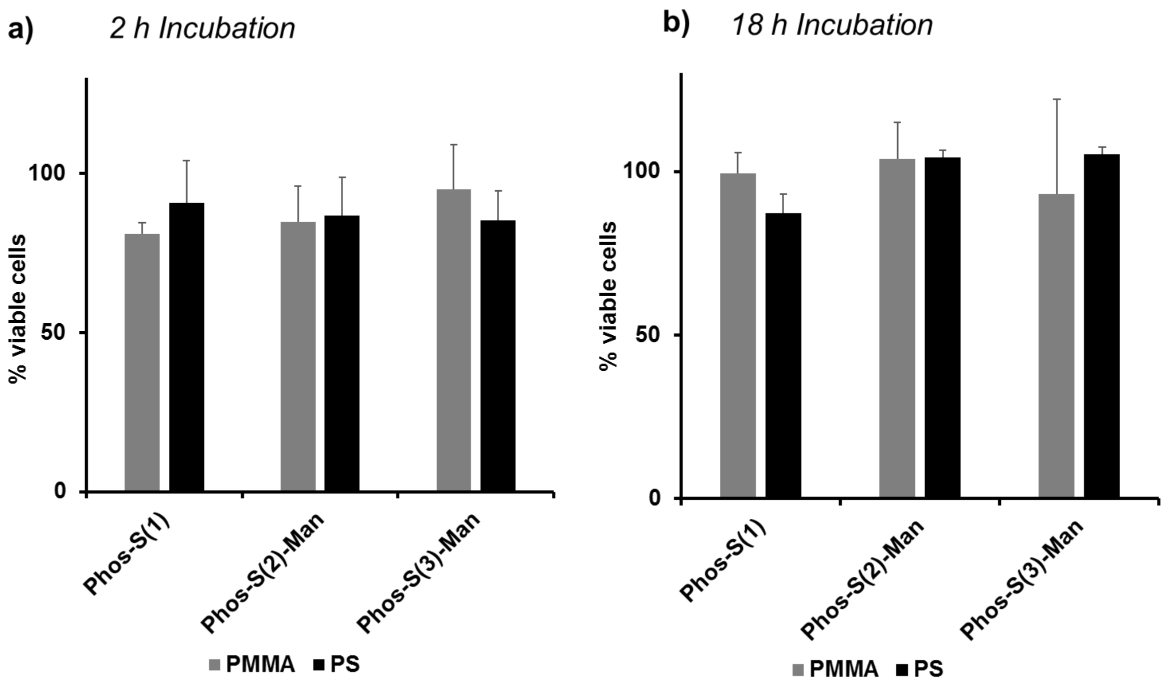

Figure S26. Monocyte derived dendritic cells were incubated with PS/PMMA nanocarriers (150 µg/mL) for 2 h or 18 h. Cells were stained with 2 µg/mL propidium iodid (PI, Sigma) for cytotoxicity. Incubation of cells without nanocarriers was set to 100% viable cells.

4. Confocal laser scanning microscopy

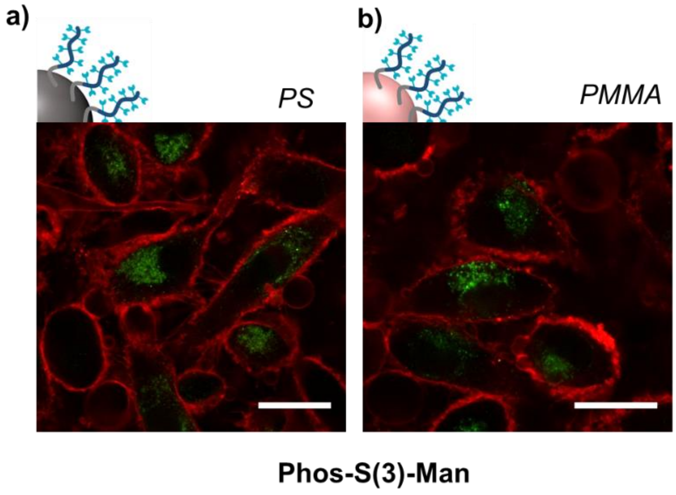

Figure S27. Interactions of PS and PMMA nanocarriers with dendritic cells. Dendritic cells were treated with Phos-S(3)-Man coated nanocarriers (150 µg/mL) for 2 h, 37 °C. The cell membrane was stained with CellMask Orange and pseudo-coloured in red whereas the nanocarriers were pseudo-coloured in green. Scale bar: 20 µm.

5. Detection of mannose on the nanocarrier surface

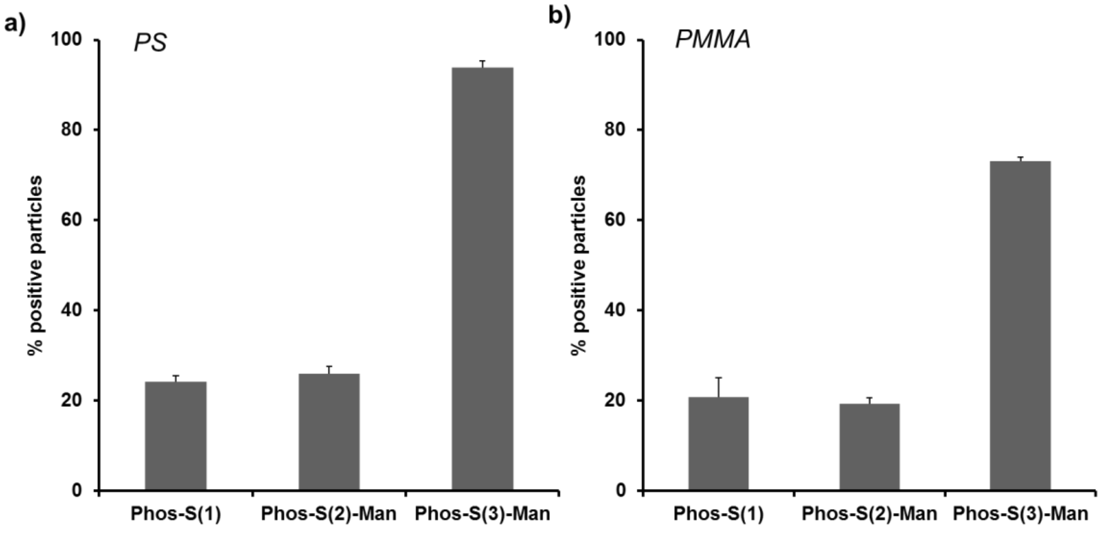

Figure S28. Nanocarriers were incubated with Alexa633 conjugated to Wheat Germ Agglutinin (WGA lectin) for 30 min at room temperature. The amount of bound WGA to mannose on the nanocarrier surface was detected by flow cytometry. Values are given in % as Alexa633 positive nanoparticles.

6. Antibody blocking

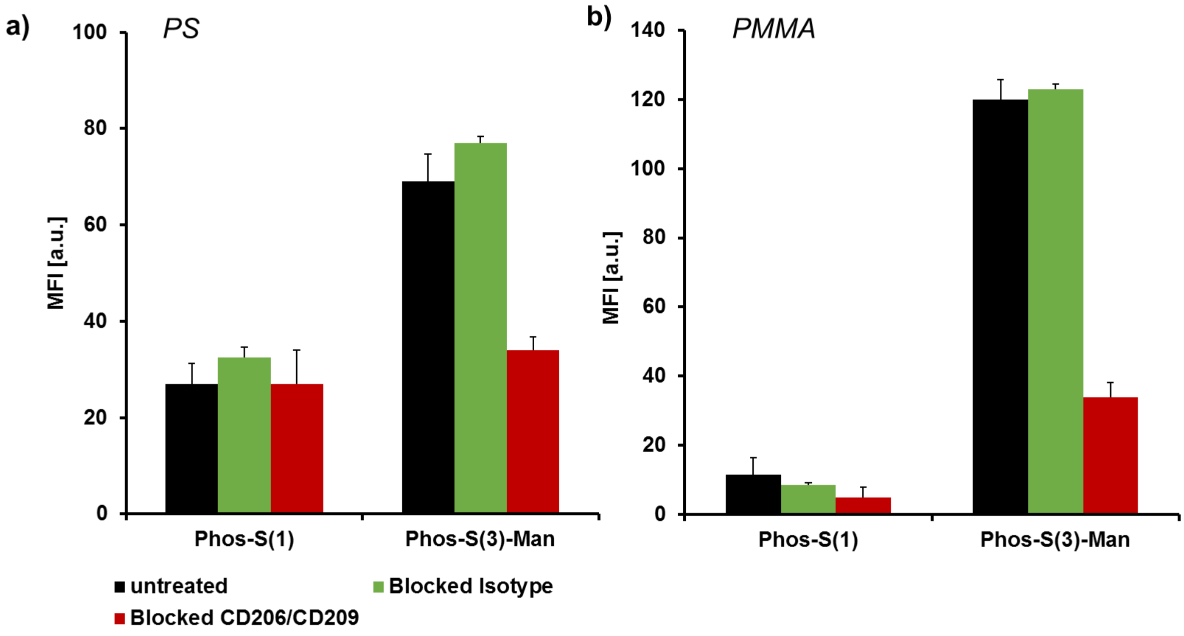

Figure S29. Interactions of PS (a) or PMMA (b) nanocarriers with dendritic cells. **a, b)** Flow cytometry analysis of Phos-S(1) or Phos-S(3)-Man coated nanoparticle. Dendritic cells were either untreated or pre-treated with different antibodies (25 µg/mL, 30 min, 4 °C) for blocking studies. Nanocarrier binding towards dendritic cells was performed at 4 °C. The median fluorescence intensity (MFI) is shown.

7. Influence of protein corona formation

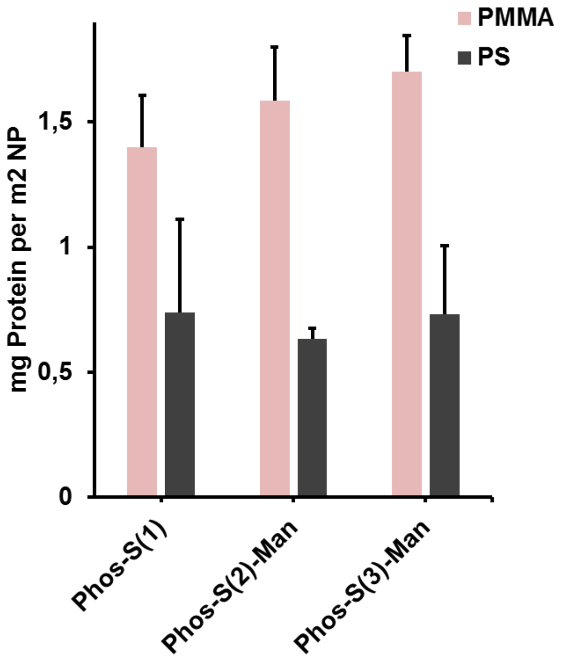

Figure S30. Pierce Assay. The amount of adsorbed corona proteins (mg) on the nanocarrier surface (m^2) after plasma incubation.

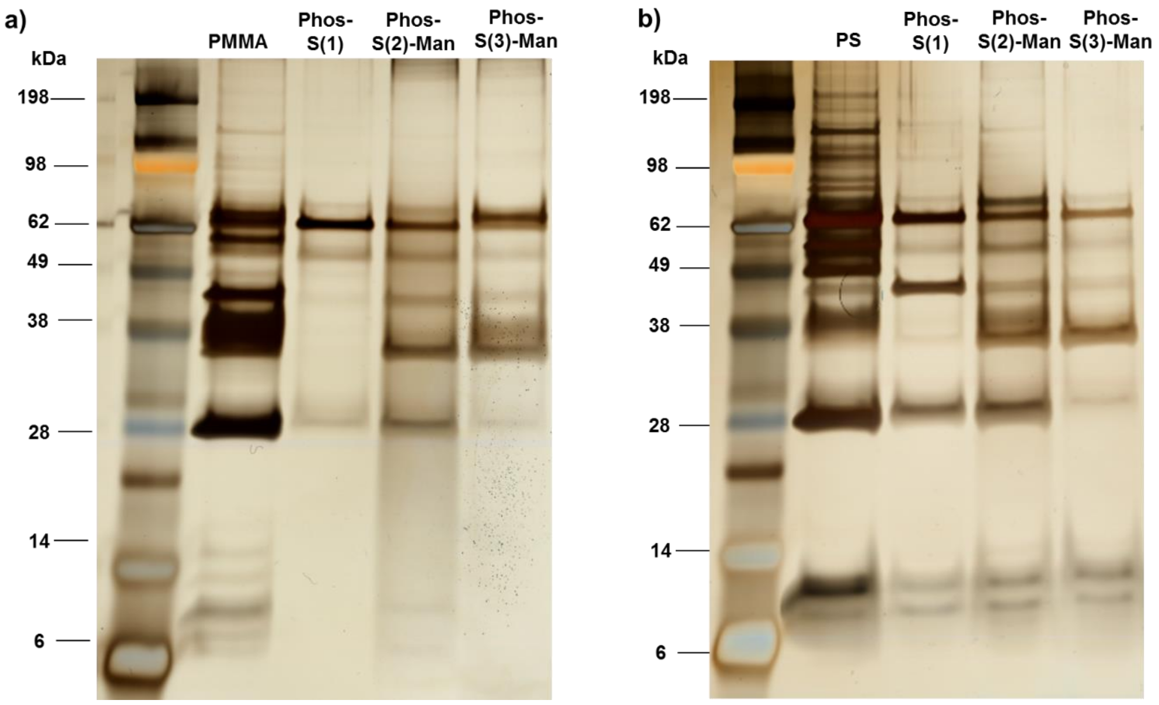

Figure S31. Unfunctionalized and surfactant coated PMMA (a) and PS (b) were incubated with human plasma for 1 h at 37 °C. The hard corona proteins were isolated as described in the material and methods section. Silver staining was used to visualize the corona pattern.

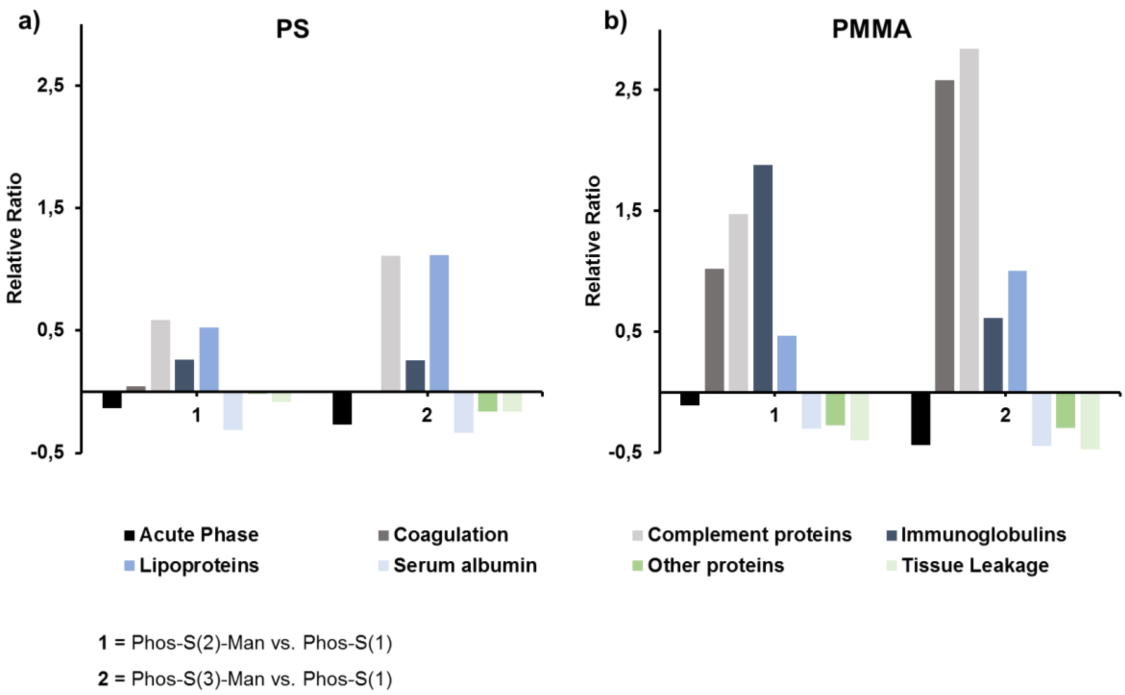

Figure S32. The hard corona proteins of PS (a) and PMMA (b) nanocarriers were analysed by LC-MS. All proteins were classified into 8 different classes depending on their biological function. The relative enrichment of proteins was calculated comparing Phos-S(2)-Man vs. Phos-S(1) or Phos-S(3)-Man vs. Phos-S(1).

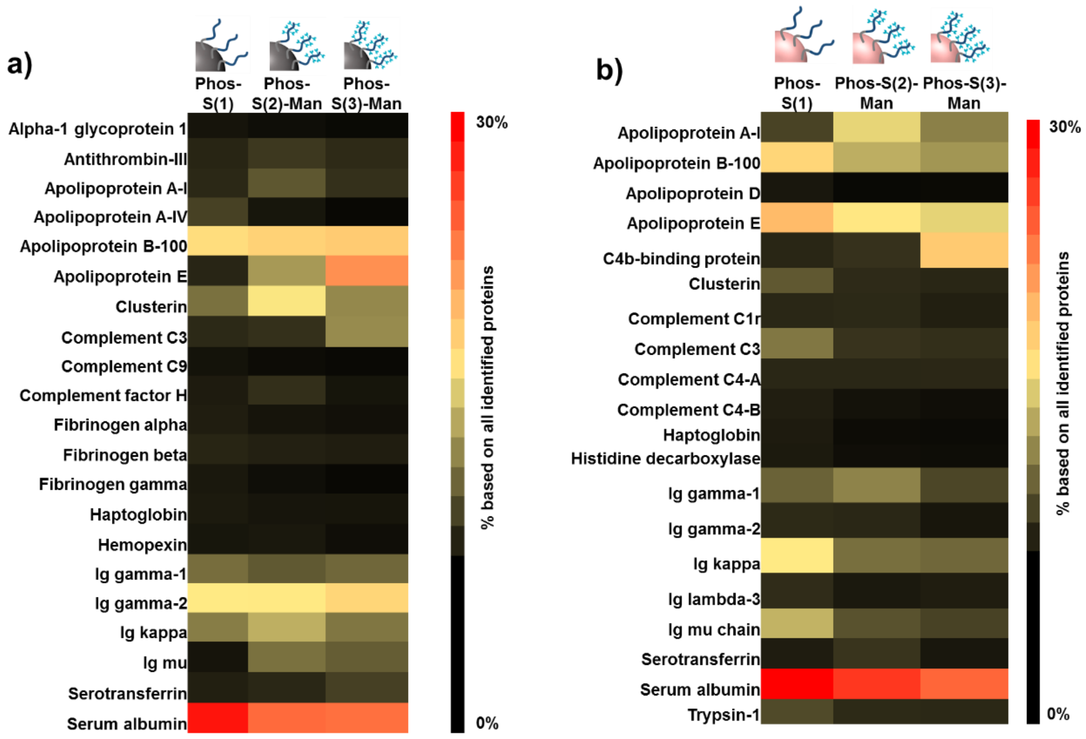

Figure S33. The hard corona proteins of PS (a) and PMMA (b) nanocarriers were analysed by LC-MS. The most abundant proteins (TOP 20) are visualized via heat map.

8. Expression level of the mannose receptor

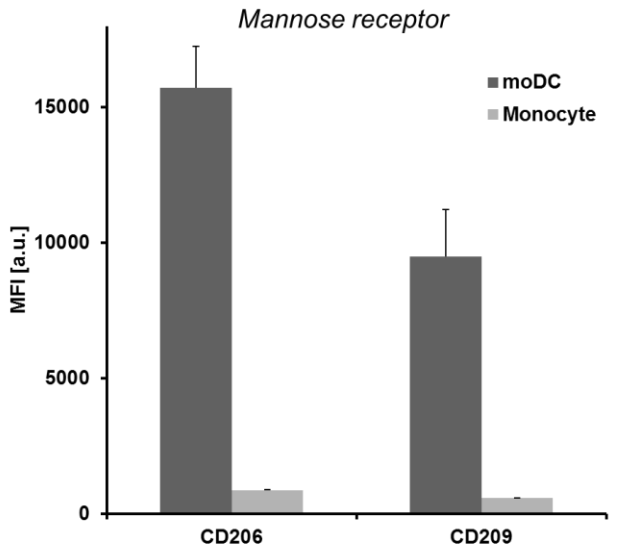

Figure S34. Monocytes or monocyte derived dendritic cells were incubated with Alexa647 labelled secondary antibodies against the mannose receptor (CD206 and CD209) for 30 min at 4 °C. Flow cytometry measurements were performed and the median fluorescent intensity values (MFI) are given.

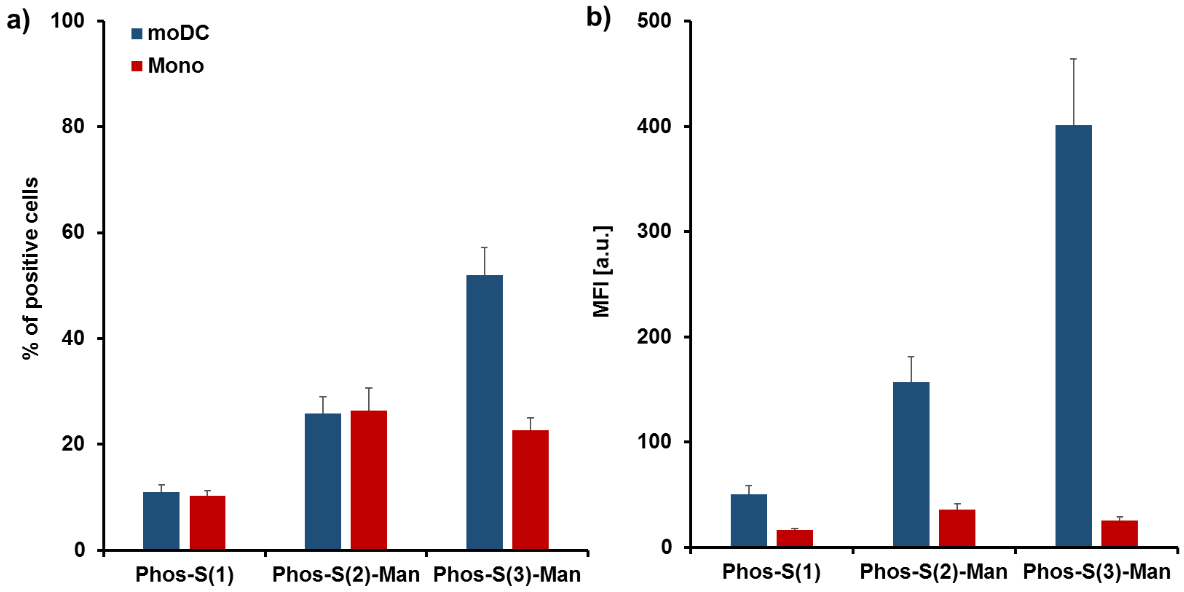

Figure S35. PS nanocarriers were exposed to human blood plasma and cellular uptake (150 µg/mL, 2 h) towards dendritic cells (blue) or monocytes (red) was quantified via flow cytometry. The amount of fluorescent positive cells in % (a) and the median fluorescence intensity (MFI) is shown (b).

7.References

[1] G. R. Fulmer, A. J. M. Miller, N. H. Sherden, H. E. Gottlieb, A. Nudelman, B. M. Stoltz, J. E. Bercaw, K. I. Goldberg, *Organometallics* **2010**, *29*, 2176-2179.

[2] E. O. Stejskal, J. E. Tanner, *The Journal of Chemical Physics* **1965**, *42*, 288-292.

[3] A. Jerschow, N. Müller, *Journal of Magnetic Resonance* **1997**, *125*, 372-375.

[4] aB. Kang, T. Opatz, K. Landfester, F. R. Wurm, *Chemical Society Reviews* **2015**, *44*, 8301-8325; bB. Kang, P. Okwieka, S. Schöttler, O. Seifert, R. E. Kontermann, K. Pfizenmaier, A. Musyanovych, R. Meyer, M. Diken, U. Sahin, *Biomaterials* **2015**, *49*, 125-134.

[5] D. Hofmann, S. Tenzer, M. B. Bannwarth, C. Messerschmidt, S.-F. Glaser, H. Schild, K. Landfester, V. Mailänder, *ACS Nano* **2014**, *8*, 10077-10088.

[6] S. Tenzer, *ACS Nano* **2011**, *5*, 7155-7167.

[7] R. A. Bradshaw, A. L. Burlingame, S. Carr, R. Aebersold, *Mol Cell Proteomics* **2006**, *5*, 787-788.

[8] M. Kokkinopoulou, J. Simon, K. Landfester, V. Mailänder, I. Lieberwirth, *Nanoscale* **2017**.

[9] J. C. Silva, M. V. Gorenstein, G. Z. Li, J. P. C. Vissers, S. J. Geromanos, *Mol. Cell. Proteomics* **2006**, *5*, 144-156.

[10] F. M. Winnik, J.-R. Brisson, J. P. Carver, J. J. Krepinsky, *Carbohydrate Research* **1982**, *103*, 15-28.
